# An intelligent approach for automated vehicle damage classification

**DOI:** 10.1038/s41598-026-61488-5

**Published:** 2026-07-20

**Authors:** Shimaa Ouf, Bassant Lotfy, Soha Ahmed

**Affiliations:** Information Systems Department, Faculty of Commerce and Business Administration, Capital University (Formerly Helwan University), Helwan, Cairo Egypt

**Keywords:** Vehicle Damage Detection, YOLOv5, YOLOv8, XGBoost, Cost Estimation, Deep Learning, Machine Learning, Engineering, Mathematics and computing

## Abstract

Manual examination using imitative assessment methods requires considerable investment in time, effort, resources, and funds. Furthermore, these assessments often exhibit inconsistencies. Therefore, this study introduces a framework for accurately detecting and classifying vehicle damage and estimating repair costs using deep learning and machine learning approaches. In this study, YOLOv5 and YOLOv8 models were applied to detect and classify vehicle damage types, such as broken lamps, glass shatters, cracks, scratches, and dents. Repair costs were predicted using the XGBoost machine learning model. The dataset used for damage detection, classification, and cost prediction was created from scratch. It was developed by identifying the types of damage and generating bounding box coordinates around damaged areas. After extracting the bounding box coordinates and damage types, additional features were incorporated. The YOLOv8 model outperformed YOLOv5 in both detecting and classifying vehicle damage, achieving a precision of 87% on the validation dataset and mean absolute precision 90.7% on the test dataset. The XGBoost model achieved an R² score of 97.28% and a Mean Absolute Error (MAE) of 128.50. These results confirm that the proposed framework enhances the precision of vehicle damage detection and classification while accelerating assessment and ensuring its effectiveness in real-world scenarios.

## Introduction

The automotive industry has become crucial, and its importance has increased. Consequently, the demand for modern systems for these industries has increased, and this trend is evident in autonomous driving and inspection systems. One of the areas that has gained significant importance is the systems used to classify, detect, and locate damage for evaluation. All these systems aim primarily to facilitate inspection processes, reduce errors caused by traditional manual evaluation, and reduce inspection time^1,2^.

Despite the rise of modern systems for detecting and classifying damage, traditional manual inspection remains prevalent in insurance companies and repair shops. This inspection method is expensive, subjective, and labor-intensive. Due to the number of defects, the desire to convert this traditional method to modern automated methods using artificial intelligence has increased to improve the precision of damage assessment^3^.

Convolutional neural networks (CNNs) have been used for the classification, detection, and identification of damage types such as scratches and cracks. These models have a great ability to learn from images for the purpose of classifying vehicle damage, especially when trained on a large dataset^4,5^.

CNNs have contributed to building a significant foundation in classification tasks, but these networks lack the ability to determine the location of damage; they only classify. This shortcoming led to a shift from relying solely on CNNs to using frameworks for object detection, namely YOLO (You Only Look Once), because it can provide algorithms that have the benefits of actual detection by predicting the bounding box as well as the type of damage for multiple types in an image^6,7^.

One of the recent contributions is the use of deep learning models that utilize multiple and diverse data angles. Examples of this include studies in which researchers suggested using three-dimensional images of vehicles because they provide a more comprehensive view of damage, and this helps in increasing the precision for detecting damage by using more than one dimension^8^. Modern systems have also been introduced that have a great ability to deal with the severity of damage and the variation between types of damage^9^.

Segmentation techniques such as R-CNN were used, which can determine the location of the damaged area with great precision. This method is considered very crucial for determining the forms of damage, especially those that are irregular in shape^10,11^.

Studies have been conducted to detect and classify vehicle damage; however, no solutions have been identified for predicting repair costs. Previous studies have recommended developing datasets that support damage detection, classification, and repair cost prediction. Accordingly, this study addresses that gap by building a dataset designed for detecting and classifying vehicle damage as well as predicting repair costs.

In this study, a novel end-to-end framework that integrates object detection, classification, and cost prediction for automated vehicle damage assessment is proposed. The framework leverages YOLO (You Only Look Once) models to accurately detect and classify diverse types of vehicle damage, followed by an XGBoost regression model to estimate repair costs.

A key contribution of this work is the creation of a custom, expert-annotated dataset, developed from scratch, which includes bounding box annotations, damage categories, severity levels, spatial locations, and corresponding repair costs. This dataset bridges the gap between visual damage detection and quantitative cost estimation, which is absent from existing research.

Furthermore, the proposed framework represents a significant advancement by combining deep learning and machine learning in a unified pipeline. Specifically, deep learning is employed for precise damage detection and classification, while regression-based machine learning is utilized for accurate cost prediction based on extracted features. This integration enables a more practical and scalable solution for real-world applications in vehicle inspection and insurance assessment.

Overall, the main contribution of this work lies in:

(1) developing a comprehensive dataset linking visual damage attributes to predict costs,

(2) designing an integrated detection, classification, and cost prediction framework,

(3) demonstrating the effectiveness of combining YOLO-based object detection with XGBoost regression for automated, data-driven vehicle damage evaluation.

## Related Work

The field of modern vehicle damage assessment has been developed significantly and rapidly by integrating deep learning models. These techniques include convolutional neural networks (CNNs) and object detection models such as YOLO and Mask R-CNN. New contributions have a significant role in improving the precision of the model, which can improve the precision of damage classification and accurately determine its type. Lee et al. used an important factor, a dataset containing images of damaged vehicles, captured from three-quarter angles of each vehicle in the dataset^8^. This contributed to improving the performance of the model because the data is different from others and tends to reflect real-world condition. Similarly, van Ruitenbeek and Bhulai used CNNs for the purpose of classifying damage in images of vehicles with damage, including scratches or fractures, and it is considered one of the basic applications in this field^12^. In the following study, Qaddour and Siddiqa proposed a method for detecting, identifying, and classifying vehicle damage. They developed a deep learning model that performs the preprocessing stage and utilizes convolutional neural networks (CNNs) techniques to achieve high precision^13^.

One of the concerns of insurance companies and repair shops is that damage cannot be detected and located in real time using these modern systems, which has prompted researchers to explore techniques used in detecting single-stage objects (YOLO). Gustian et al. and later Ramazhan et al. demonstrated the effective use of YOLO models in real-time applications by detecting damage at high speeds with remarkable precision^14,15^.

Dolhopolov et al. conducted a comparative study between the latest YOLO versions (v8, v9, v10), and the difference between the versions appears in the detection precision. Therefore, YOLOv10 outperforms the previous versions, especially mAP@0.5 and F1-Score. It was noted that a small dataset was used, and they also paid attention to the diversity of the dataset, but this dataset required expansion and development^16^.

Chua et al. presented how deep learning and image classification can be applied in the insurance claims and management industry to identify and evaluate damaged vehicle parts. In auto insurance claims, appraisers are typically required to determine the extent of vehicle damage^17^.

Dwivedi et al. worked on the issue of classifying and detecting vehicle damage so that insurance firms could automate the process of filing claims. This study employed models pre-trained on a varied dataset because their dataset was very tiny, but unlike what was done in this study, this study did not build a dataset from scratch^18^.

Thar et al. suggested a convolutional neural network (CNN), and You Only Look Once v5 (YOLO v5) model-based vehicle damage volume level identification system. However, the proposed algorithm in this study was not implemented or experimentally validated. The authors stated that collecting and preparing large image datasets would require considerable time and effort. This limitation affects the practical applicability of the study, as the performance of the proposed approach was not empirically evaluated^19^.

The literature review’s conclusions show that several studies focused on the importance of creating real datasets as part of potential future research avenues. The creation of a trustworthy dataset has become crucial for improving associated computational models since there are few publicly accessible datasets that sufficiently enable vehicle damage detection, classification, and repair cost prediction. To close this gap and make future research and model-building easier, the suggested methodology builds a complete dataset to detect and classify damage and predict the repair cost. The key findings and focus areas of previous studies are summarized, as presented in Table 1.

**Table 1:** Summary of Previous Studies.

Research	Year	Model	Dataset Size	Evaluation
^15^	2025	YOLOv9	4,000	Precision = 0.780 Recall = 0.690 mAP@0.5 = 0.730 mAP@0.5:0.95 = 0.580 F1-Score = 0.7322
^16^	2025	YOLOv8	7,258	Precision = 0.6335 Recall = 0.55621 mAP@0.5 = 0.59692 mAP@0.5:0.95 = 0.30884 F1-Score = 0.59246
		YOLOv9	7,258	Precision = 0.655 Recall = 0.573 mAP@0.5 = 0.630 mAP@0.5:0.95 = 0.333 F1-Score = 0.619
		YOLOv10	7,258	Precision = 0.675 Recall = 0.624 mAP@0.5 = 0.651 mAP@0.5:0.95 = 0.349 F1-Score = 0.649
^18^	2021	YOLOv3 (416 × 416)	11,554	Precision = 0.86 Recall = 0.70 mAP@0.5 = 0.7423 F1-Score = 0.77
		YOLOv3 (608 × 608)	11,554	Precision = 0.70 Recall = 0.80 mAP@0.5 = 0.7778 F1-Score = 0.74
^20^	2023	DCN+	9,000	mAP = 0.606
^21^	2024	YOLOv5	13,945	mAP@0.5 = 0.484
		Mask-RCNN X101-FPN	13,945	mAP@0.5 = 0.596
		YOLOv3	13,945	mAP = 0.378
^22^	2022	SipMask++	1032	mAP = 0.720
		Yolact	1032	mAP = 0.665
^23^	2022	DenseNet-169	N/V	Precision = 0.81
		VGG-19	N/V	Precision = 0.78
^24^	2024	YOLO	2508	Precision = 0.785 Recall = 0.7024 mAP = 0.66
^25^	2021	VGG19	51,770	Precision = 50%
		MobileNet	51,770	Precision = 70%

At the end of previous studies, it was noted that all these studies were only concerned with detecting or classifying the damage and ignored predicting the cost of the damage, unlike what our proposed study, it detects and classifies the damage and predicts the cost of repairing damage. Another flaw in the studies^12,14–16,26,27^ was that they relied on an unbalanced dataset. The proposed study relied on a more balanced and accurate dataset. This study did not rely on a previous dataset but rather worked to build a dataset specifically for detecting, classifying, and predicting the cost of vehicle damage. To get rid of this problem, this study performed pre-processing to complete the tasks of balancing between categories, as well as to prove the importance of data balance. The YOLOv8 and YOLOv5 models were used on balanced and unbalanced data for detection and classification. Then XGBoost will be used to predict damage costs.

## Proposed Framework

**Fig. 1:**
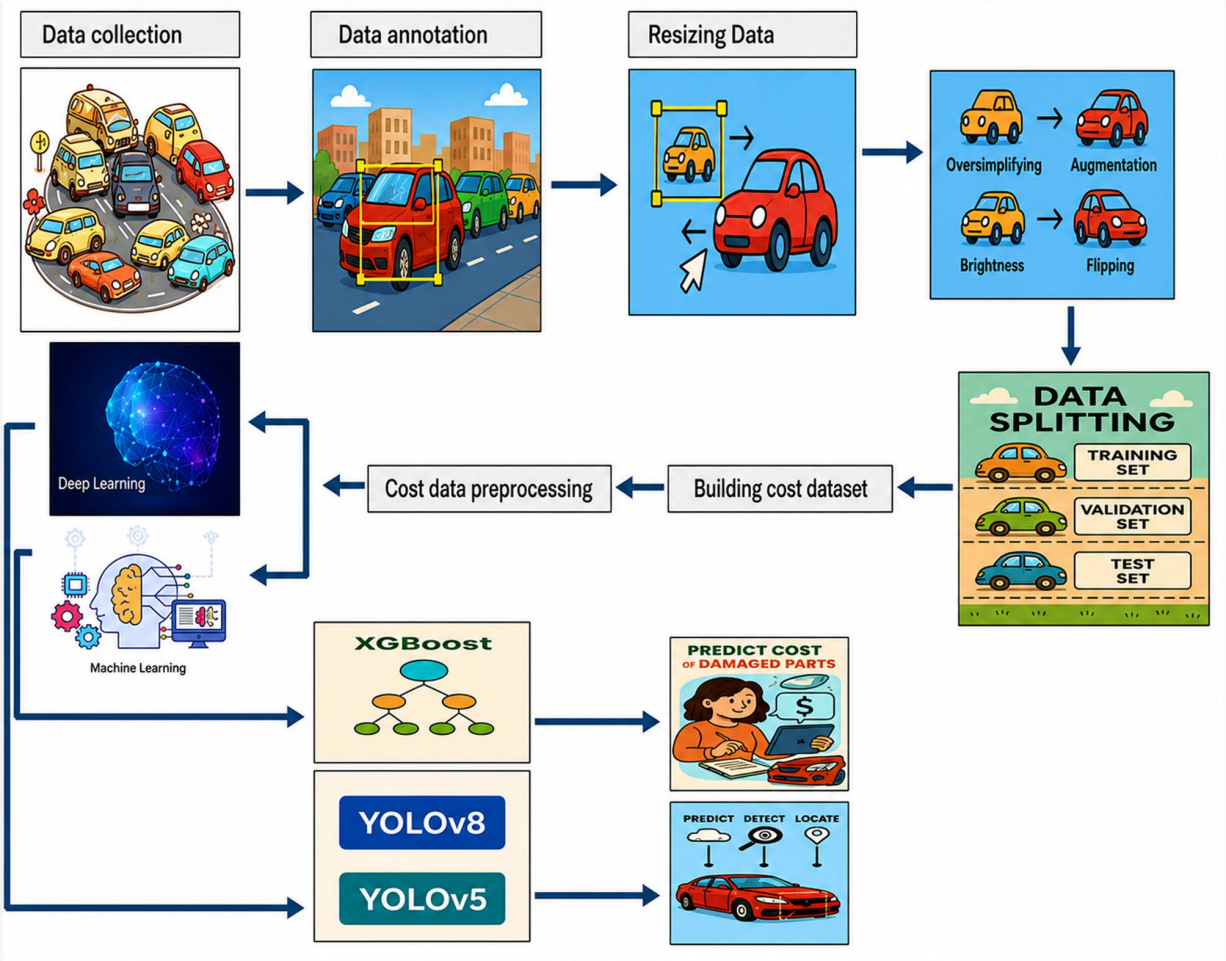
The Proposed Framework for Vehicle Damage Detection and Repair Cost Prediction.

This section will address stages to overcome the detection, classification, and cost prediction dataset’s limitations based on earlier research. There are nine primary stages in the suggested framework for improving the automotive industry: stage 1:data collection, stage 2: data annotation, stage 3: resizing data, stage 4: oversampling and data augmentation, stage 5: data splitting, stage 6: building the cost dataset, stage 7: cost data preprocessing, stage 8: deep learning models for detecting and classifying damage, stage 9:machine learning models for cost prediction. The suggested framework is an integration of deep learning and machine learning techniques. YOLOv5 and YOLOv8 deep learning models are employed for object recognition and categorization. Both an unbalanced and a balanced dataset will be subjected to the YOLOv5 and YOLOv8 models. To identify and categorize vehicle damage into five types, these models produce outputs that include the damage type and bounding box coordinates surrounding the damage area. After these attributes are retrieved, other metadata—such as location, repair cost, and damage severity—will be added. These attributes then offer an XGBoost regression model to predict the repair cost, as presented in Fig. 1.

### Data collection

The data collection stage deals with how to overcome the limits caused by the lack of datasets that will be utilized for cost prediction, detection, and classification. At this point, gathering data—not just any data, but high-quality data—is the main objective. Images of vehicles with diverse types of damage make up the data that is gathered to use deep learning and machine learning to detect, classify, and predict costs. Only image-containing files are available in the open source; the damage annotation files are not included with the photographs. As a result, creating a fresh dataset from the start was required. Five categories of damage—dent, crack, scratch, broken lamp, and glass shatter—were used for the study to detect and classify damage. To prevent the model’s outcomes from being impacted and to guarantee an impartial and efficient assessment, it was essential to make sure the photos were of high quality. These unprocessed photos were gathered via Kaggle^28^. Dent, crack, scratch, broken lamp, and glass shatter are the five features seen in these 6666 records of raw pictures. As a result, manual annotation files utilizing bounding boxes and damage types must be included and explained. This is essential for the duties of damage detection, damage categorization, and repair cost estimation. A cost dataset will be constructed using these annotation files, and further metadata will be added.

### Data annotation

Bounding boxes must be manually positioned in every image to indicate the damage area on the vehicles to effectively train, verify, and evaluate the model for damage recognition, classification, and cost prediction. Dents, cracks, scratches, broken lamps, and glass shatters were among the damage types found. Bounding boxes were placed on all the photographs using tools because the dataset was created from scratch. Many types of damage and, hence, many bounding boxes may be present in a single picture. This is to guarantee that the model detects items accurately. The extension of the annotation files was exported so that they could be used with any model.

### Resizing data

The size of the pictures used to train, validate, and test deep learning models must be adjusted to be compatible with the models to conduct the preprocessing procedure on the images. This phase is crucial to improving the whole dataset’s compatibility with one another. This guarantees the precision of the models, particularly those that identify objects with ease.

Three typical sizes are taken into consideration while considering this phase.

- 416 × 416: This size has the benefits of using less memory and offering quicker processing. Although it is ideal for real-time processing, the tiny size of the image negatively impacts detection precision, particularly in cases of minor damage.
- 800 × 800: This has the benefit of maintaining detecting precision because of the image’s enormous size, but it also uses more memory, takes longer to analyze, and takes longer to train.
- 640 × 640: This size is an excellent option as it strikes a balance between efficiency in terms of time and resources needed for the detection process and size.

### Oversampling and Data Augmentation

This stage will discuss how to address the issue of unbalanced datasets at this point. Oversampling and data augmentation were the best ways to avoid overfitting because deep learning was used for detection and classification.

**Oversampling**.

When processing damage types that are thought to be less common than other types in the dataset, the best course of action in deep learning is to use an oversampling strategy to enhance their quantity.

The original dataset was imbalanced, with the Scratch Damage class accounting for the largest proportion of samples (48.20%) compared to the other classes. To prevent model bias and ensure high detection accuracy for minority classes such as broken lamps and glass shatter, oversampling and data augmentation were applied.

Before balancing, the training set showed a large variance in class distribution. For example, the Scratch class contained 4,117 instances, whereas smaller classes such as Broken Lamp had only 726 instances.

After balancing, as shown in Table 2, a more uniform class distribution was achieved. For instance, the Broken Lamp class increased to 2,695 instances, while the Glass Shatter class increased to 3,003 instances.

**Table 2:** Dataset construction before and after balancing.

Damage Type	Data before balancing	Data after balancing
Dent	886	3,594
Crack	1,081	3,200
Scratch	4,117	3,470
Broken Lamp	726	2,695
Glass Shatter	844	3,003
Total	**8**,**541**	**15**,**962**

#### Data augmentation

The dataset is effectively improved by data augmentation. This is accomplished by altering the datasets in a way that will enhance the model, lessen overfitting, and increase the model’s performance under ideal circumstances.

#### Brightness adjustment

This phase involves adjusting brightness to suit various lighting situations. This lessens the model’s sensitivity to various situations by allowing it to function in them. Performance is enhanced as a result, particularly during training.

#### Flipping horizontally

The model’s capacity to detect and classify damage is enhanced by horizontal flipping, which broadens the variety of datasets and enhances damage direction variability. Although horizontal flipping of photos minimizes overfitting that arises from the repetition of the forms of damage that are intended to be enhanced, this step is crucial for expanding the diversity of data.

### Data Splitting

Particularly for damage detection, classification, and cost prediction tasks, data splitting is a critical step in increasing the precision of deep learning models. As a result, the dataset was split into three sections for this study: training, validation, and testing. When it comes to damage detection and classification, this approach is crucial. To enable the model to detect and classify damage, labels and annotation files are learnt during the training phase. The learning stage is enhanced while the model results are displayed following each validation epoch during the validation stage. The model is then evaluated and its capacity to generalize to a new dataset is assessed during the testing phase, which is distinct from the training and validation phases.

### Building the cost dataset

For the machine learning model to correctly predict the repair cost for each type, the dataset of damage repair prices had to be constructed using a dependable approach due to the lack of cost datasets. It would have been better to speak with a vehicle repair specialist. This was relied upon because the five damage categories mentioned in this study—glass breaking, broken lights, scratches, fractures, and dents—do not have an open-source database that includes repair prices. As a result, the dataset was constructed from the ground up. This is to guarantee that it is accurate and consistent with the other damage detection and classification operations. To ensure integration between the two activities, the cost dataset was created by allocating a cost to every picture used for damage detection and classification tasks. At this point, the location, severity, and cost characteristics will be combined with the information from the annotation files. The characteristics of the cost dataset include damage type, damage severity (low, medium, high), damage location (front, side, rear), and bounding box coordinates (x_center, y_center, width, height).

### Cost Data Preprocessing

In this stage, the dataset cost is created and processed to ensure that the regression model is the best to predict the cost, and the data is consistent with each other. The processing stage includes three steps: Step one: eliminate any duplicate records. Step two: check missing values. The last step: Label Encoding, in which all textual features will be converted into numerical representations.

### Deep Learning models

Deep learning models are employed in this stage to detect and classify image damage, namely dent, crack, scratch, broken lamp, and glass shatter once the dataset preparation and pre-processing are finished. They will be trained in images with annotation files in the TXT annotation format that provide bounding boxes and damage types. This made it simple for the model to classify damage types and detect characteristics using the bounding box coordinates.

#### YOLOv5

YOLOv5 is a computer vision object detection model. It performs effectively for real-time jobs because of its rapid inference speed and enhanced functionality over previous YOLO models. YOLOv5 comes in a variety of forms and is quite good at perceiving tiny details. After YOLOv4 was released, Ultralytics unveiled YOLOv5, which was far better than previous YOLO models. YOLOv5 is used in different jobs and yields dependable results, even though there are several disagreements over its legality since it was not published as peer-reviewed research. For inference, it operates at 140 frames per second. YOLOv5 uses PyTorch, which increases the speed and precision of model deployment. YOLOv5 has now surpassed YOLOv4 in a number of instances, even though the frameworks of YOLOv4 and YOLOv5 are so similar that it is hard to assess their differences. There are five sizes available for the YOLOv5 model: nano, small, medium, huge, and extra-large. The model type is ascertained using the dataset. The lightweight YOLOv5 model is also available in version 6.0, which boasts an improved inference speed^29^.

#### YOLOv8

For instance, segmentation, image identification, and object detection tasks, the most recent YOLO model, YOLOv8, is appropriate. YOLOv8 was created by the same business that created the YOLOv5 model. YOLOv8 includes several improvements and changes to the architecture and development experience over YOLOv5. You Only Look Once, or YOLO, models have become well-known in the field of computer vision. YOLO’s outstanding precision and compact model size are a result of its popularity. YOLO models may be used by a range of developers since they can be trained with a single GPU. Deep learning practitioners can deploy it on edge hardware or the cloud at a low cost^30^.

#### YOLO Model Architecture

Convolutional neural network-based models for real-time object recognition are known as “You Only Look Once,” or YOLO. According to the paper, it transformed object identification by treating the issue as a single regression job to detect and classify damage and by utilizing whole pictures to predict bounding boxes and class probabilities in a single run, as presented in Eq. 1^16^.

1 $$\:\boldsymbol{Y}=\boldsymbol{F}\:(\boldsymbol{X};\:\boldsymbol{\theta\:})$$

Where:

X represents the input picture.

F is the YOLO model function.

θ represents the model parameter.

Y is the output prediction, which includes class probabilities and bounding boxes.

#### Evaluation Metrics of all YOLO Models

Performance metrics are essential instruments for assessing the precision and effectiveness of object identification algorithms. They provide insight into how well a model can detect and classify items in pictures. They also aid in comprehending how the model oversees false positives and false negatives.

- **IOU**,** or Intersection over Union**: It has a range of [0 to 1] and is determined by dividing the sum (union) of A and B by the intersection area of the predicted segmentation map A and the reality on the ground map B, as presented in Eq. 2^13^.

2 $$\:\mathbf{I}\mathbf{o}\mathbf{U}\:=\:(\mathbf{A}\:\cap\:\:\mathbf{B})\:/\:(\mathbf{A}\:\cup\:\:\mathbf{B})\:$$

Where:

The Mean of IOU is the mean IOU for every class.

The following equations will be used to determine precision, recall, and F1 score for each class as well as overall:

- **The precision (P).** The metric quantifies the proportion of correctly identified items, as presented in Eq. 3^13^.

3 $$\:\mathbf{P}\boldsymbol{r}\boldsymbol{e}\boldsymbol{c}\boldsymbol{i}\boldsymbol{s}\boldsymbol{i}\boldsymbol{o}\boldsymbol{n}\:=\:\boldsymbol{T}\boldsymbol{P}\:/\:(\boldsymbol{T}\boldsymbol{P}\:+\:\boldsymbol{F}\boldsymbol{P})\:\:\:\:\:\:\:\:$$

Where:

TP stands for True Positives.

False Positives (FP).

- **Recall**^®^ is the ability to precisely find each relevant instance (object) in a dataset. In other words, it calculates the percentage of real positive examples, also known as ground truth objects, that the YOLO model correctly identified, as presented in Eq. 4^13^.

4 $$\:\:\:\mathbf{R}\mathbf{e}\mathbf{c}\mathbf{a}\mathbf{l}\mathbf{l}\:=\:\mathbf{T}\mathbf{P}\:/\:(\mathbf{T}\mathbf{P}\:+\:\mathbf{F}\mathbf{N})\:$$

Where:

FN stands for False Negatives.

- **The F1-score**, which is the harmonic mean of precision and recall, is a fair assessment of a model’s performance that accounts for false negatives as well as false positives, as presented in Eq. 5^13^.

5 $$\:\mathbf{F}1\:=\:2\:\times\:\:(\mathbf{P}\mathbf{r}\mathbf{e}\mathbf{c}\mathbf{i}\mathbf{s}\mathbf{i}\mathbf{o}\mathbf{n}\:\times\:\:\mathbf{R}\mathbf{e}\mathbf{c}\mathbf{a}\mathbf{l}\mathbf{l})\:/\:(\mathbf{P}\mathbf{r}\mathbf{e}\mathbf{c}\mathbf{i}\mathbf{s}\mathbf{i}\mathbf{o}\mathbf{n}\:+\:\mathbf{R}\mathbf{e}\mathbf{c}\mathbf{a}\mathbf{l}\mathbf{l})\:$$

### Machine learning model

After completing the task of detecting and classifying damage using deep learning models, the next stage will be predicting the cost of repairing damage using machine learning models. Machine learning models were built using data containing numerical and detection and classification features, namely the type of damage, its location, severity, and bounding box coordinates. All these features helped improve the model’s performance. The model will be trained on the training dataset, and to ensure its applicability in the real world, the performance was evaluated using the validation dataset. All of this contributed to building a model that could provide accurate evaluations and predictions.

#### XGBoost

The Extreme Gradient Boosting (XGBoost) model was chosen due to its efficiency and ability to oversee numerical and categorical features. Gradient boosted decision trees, a supervised learning boosting approach that leverages gradient descent, are used by the distributed, open-source machine learning package XGBoost (extreme Gradient Boosting). It is renowned for being quick, effective, and scalable when working with big datasets. The XGBoost model may be expressed mathematically, as presented in Eq. 6^31^.

6 $$\:\mathbf{y}^\mathbf{}\mathbf{i}\mathbf{}={\sum\:}_{\mathbf{k}=1}^{\mathbf{K}}\:\:{\mathbf{f}}_{\mathbf{k}}\left({\mathbf{x}}_{\mathbf{i}}\right)\:$$

Where:

y^i is the final predicted value for the ith data point.

K is the ensemble’s tree count.

fk(xi) depicts the predict of the Kth tree for the ith data point.

## Implementation and results

The actual implementation of the proposed framework in each stage will be demonstrated step-by-step in this section, along with more information on the features and strategies employed in each step and the outcomes generated for each stage, all of which will be thoroughly explained.

### Data collection

**Fig. 2:**
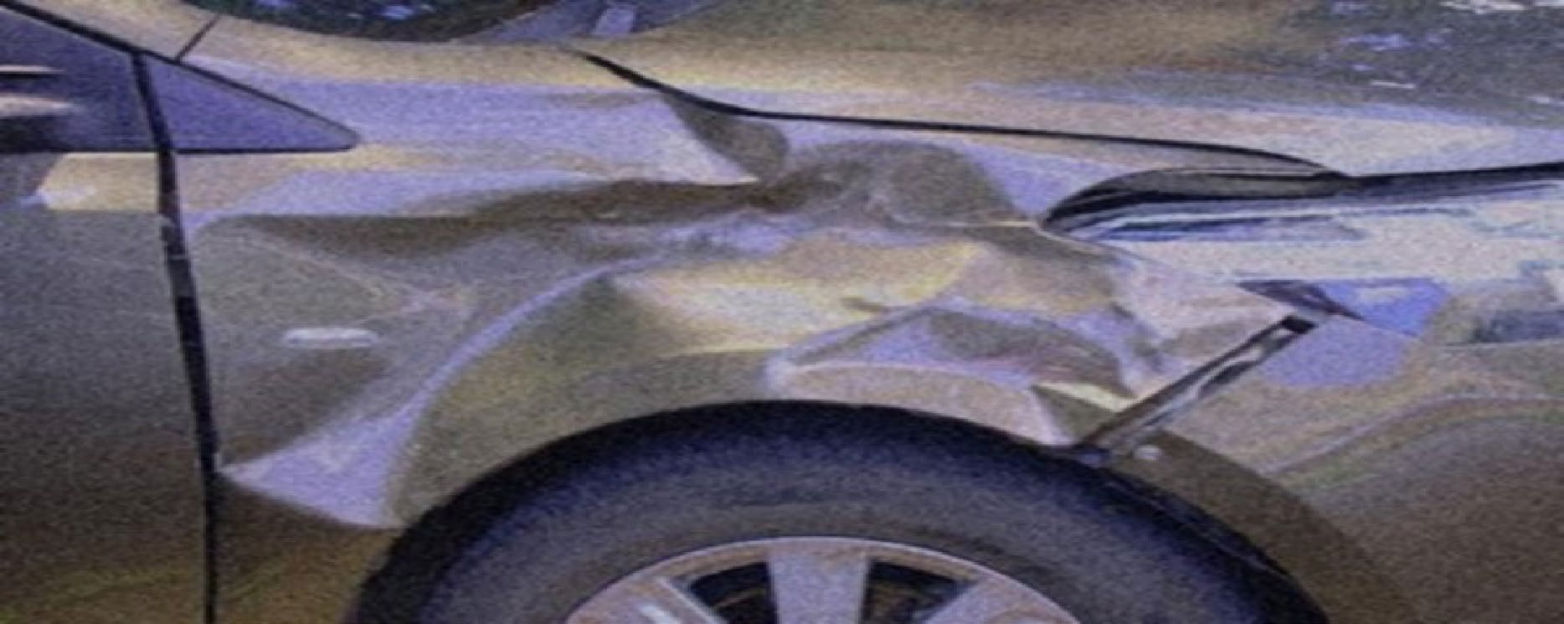
Raw image obtained from Kaggle and has no annotations.

The first stage in putting the proposed framework into practice is gathering and refining the data set to make it more fit and suitable for the study tasks—namely, detecting and classifying vehicle damage. Only pictures of damaged vehicles without any annotation files attached were discovered during the initial stage of looking via free sources, particularly Kaggle^28^. There were only images of damaged vehicles without any annotation files attached to them. These images contained 6666 records and five features: dent, crack, scratch, shattered light, and glass shatter. This dataset, which consists of images of damaged vehicles, was created for the purpose of classification, not for the purpose of detecting damage. Due to the lack of complete integration and availability of this data, only images without annotation were found. Due to these limitations, which are due to the lack of detailed information about the location of the damage, represented by the type of damage and the coordinates of the bounding boxes, which are a basic requirement for training detection models, it was necessary to build this dataset from scratch. Raw image without any annotation file is presented in Fig. 2.

### Data annotation

**Fig. 3:**
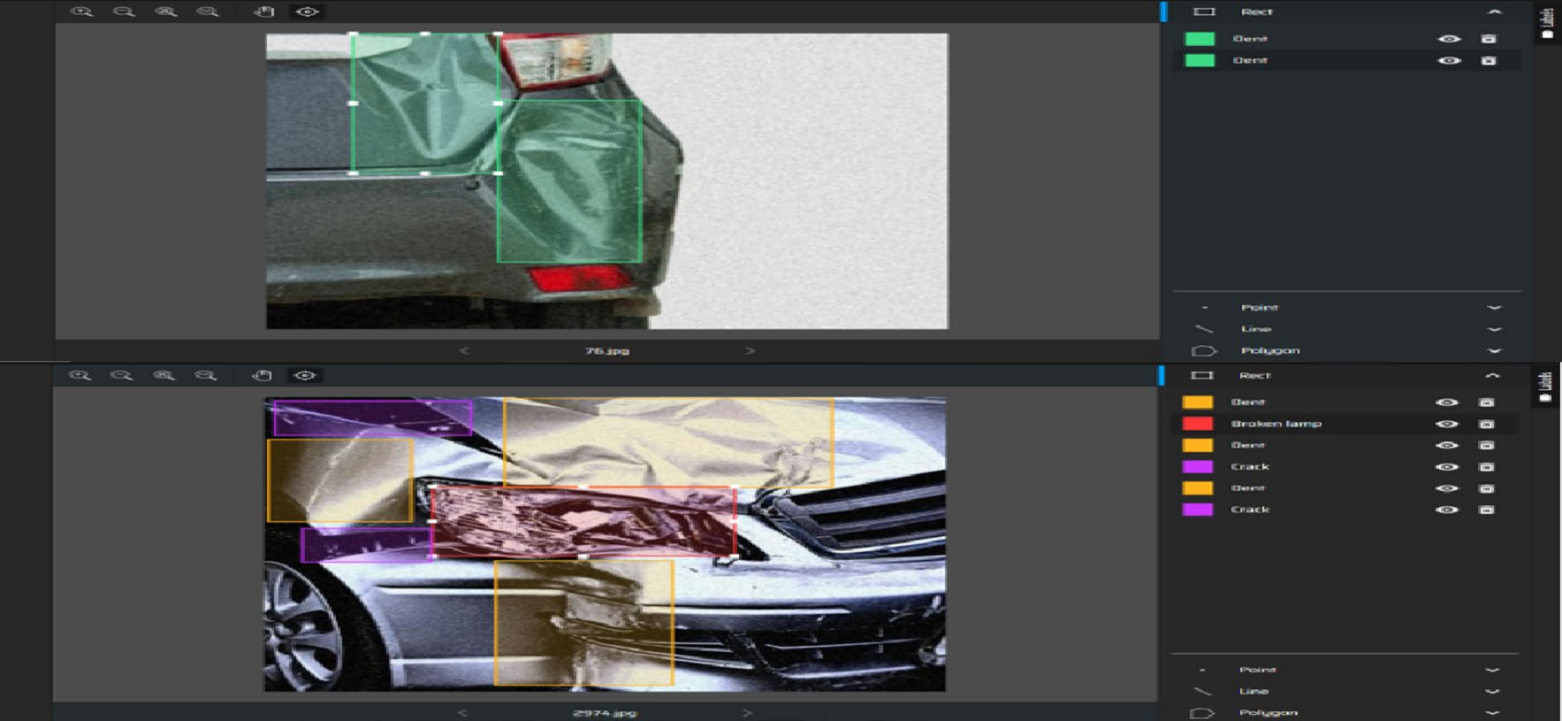
demonstrates how the website is used to create bounding boxes.

The type of damage and the bounding box coordinates must be added as annotations so that the deep learning model can find the damage properly. The annotation procedure had to begin from nothing because the previous dataset only comprised pictures of automobiles with damage. The primary processes were as follows:

• Manual bounding box drawing procedure: Every picture of a damaged vehicle was scrutinized to manually construct bounding boxes around each damage. To detect the affected regions, this phase is crucial.
• Damage classification stage: Each damage type is identified and categorized in this step, with five categories: dent, crack, scratch, broken lamp, and glass shatter. Because of this categorization, the object detection model can detect and classify the item.
• How to import all this data: To make it compatible with deep learning models used for damage detection and classification, this data was exported in the YOLO format, which has a TXT extension. Every picture file had a TXT extension that included the following information: class_id, x_center, y_center, width, and height, as presented in Fig. 3.

**Fig. 4:**
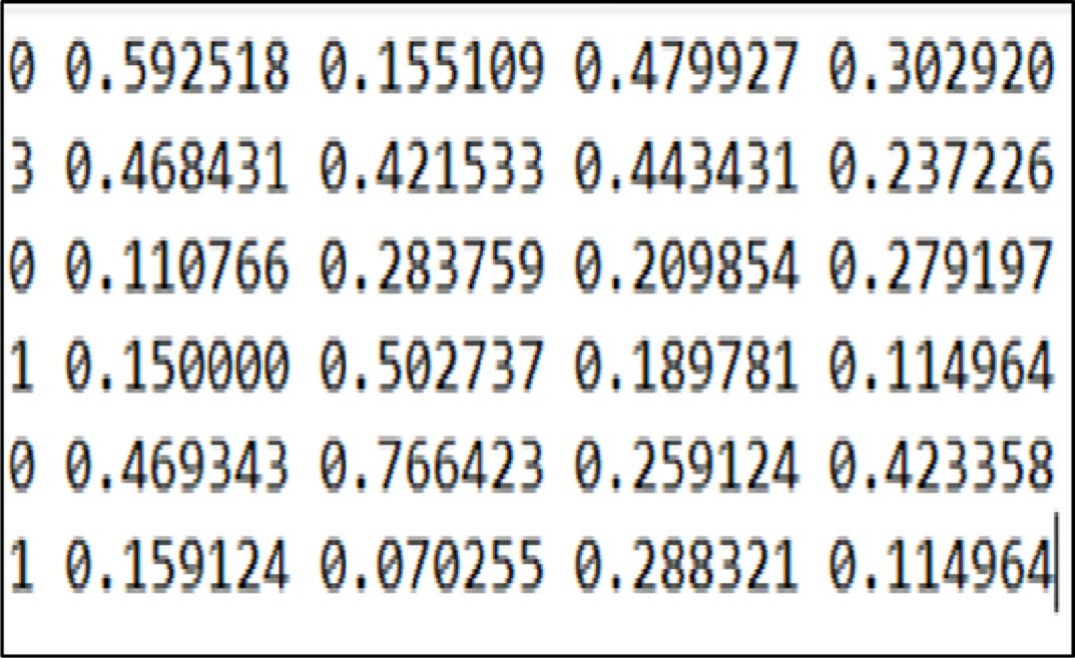
Illustrates the YOLO. txt file format after creating the bounding boxes.

The model was trained once all this data had been prepared. It was found that there were discrepancies in the annotation, and the model’s performance did not match the data set during the first training phase. To prevent past mistakes, the datasets are regenerated from the start for every image, ensuring that the labels for every form of damage are in the same sequence. It must be acknowledged that this procedure required a significant amount of time and effort, but it was essential to work on it precisely to guarantee the model’s precision when it came to detecting and classifying damage, as presented in Fig. 4.

**Fig. 5:**
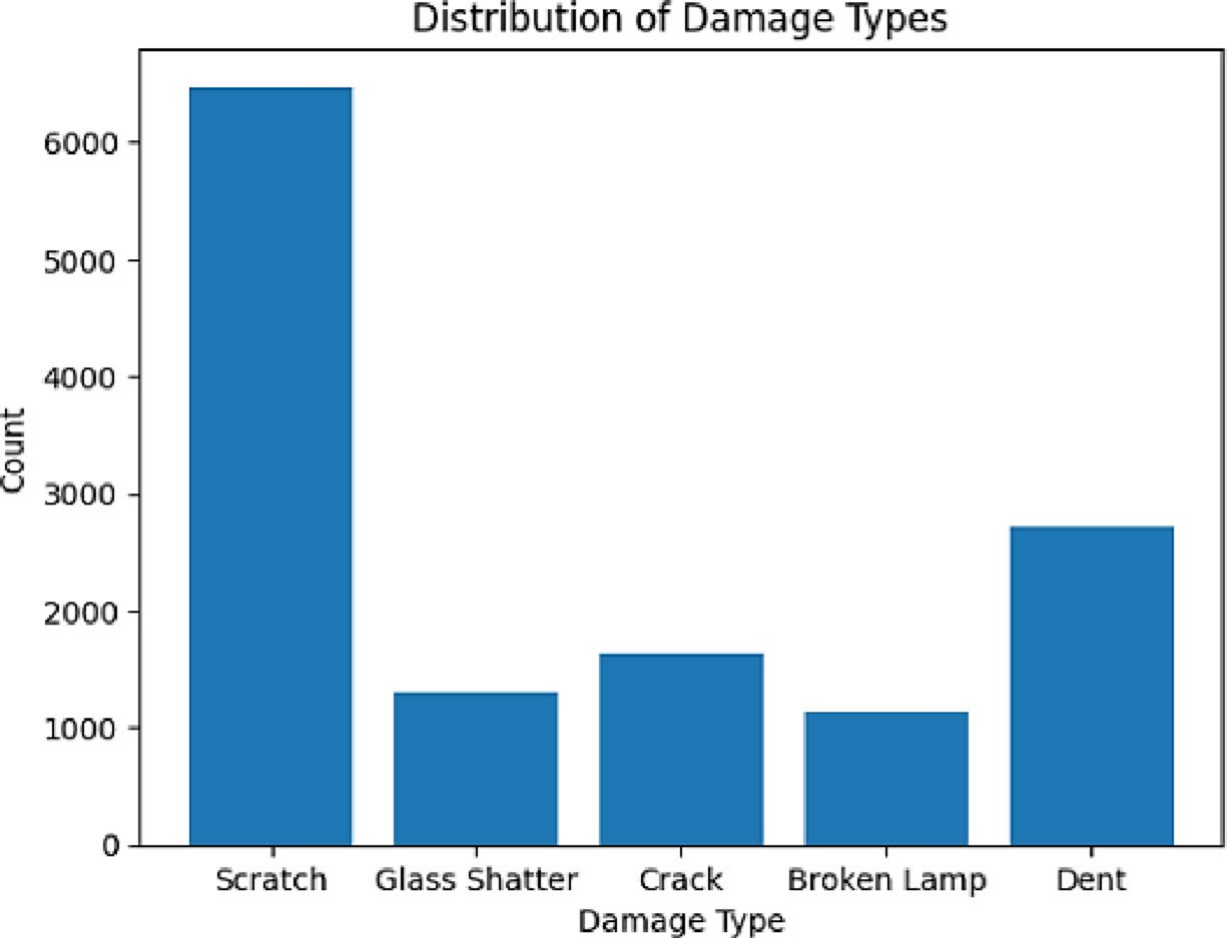
Illustrates the original distribution of the data (Imbalanced dataset).

After the annotation process was finished and bounding boxes were created, a study was conducted to ascertain the distribution of the five damage categories. It was noted that the data set was frequently out of balance since the scratch category had the most photos, as presented in Fig. 5.

### Resizing data

**Fig. 6:**
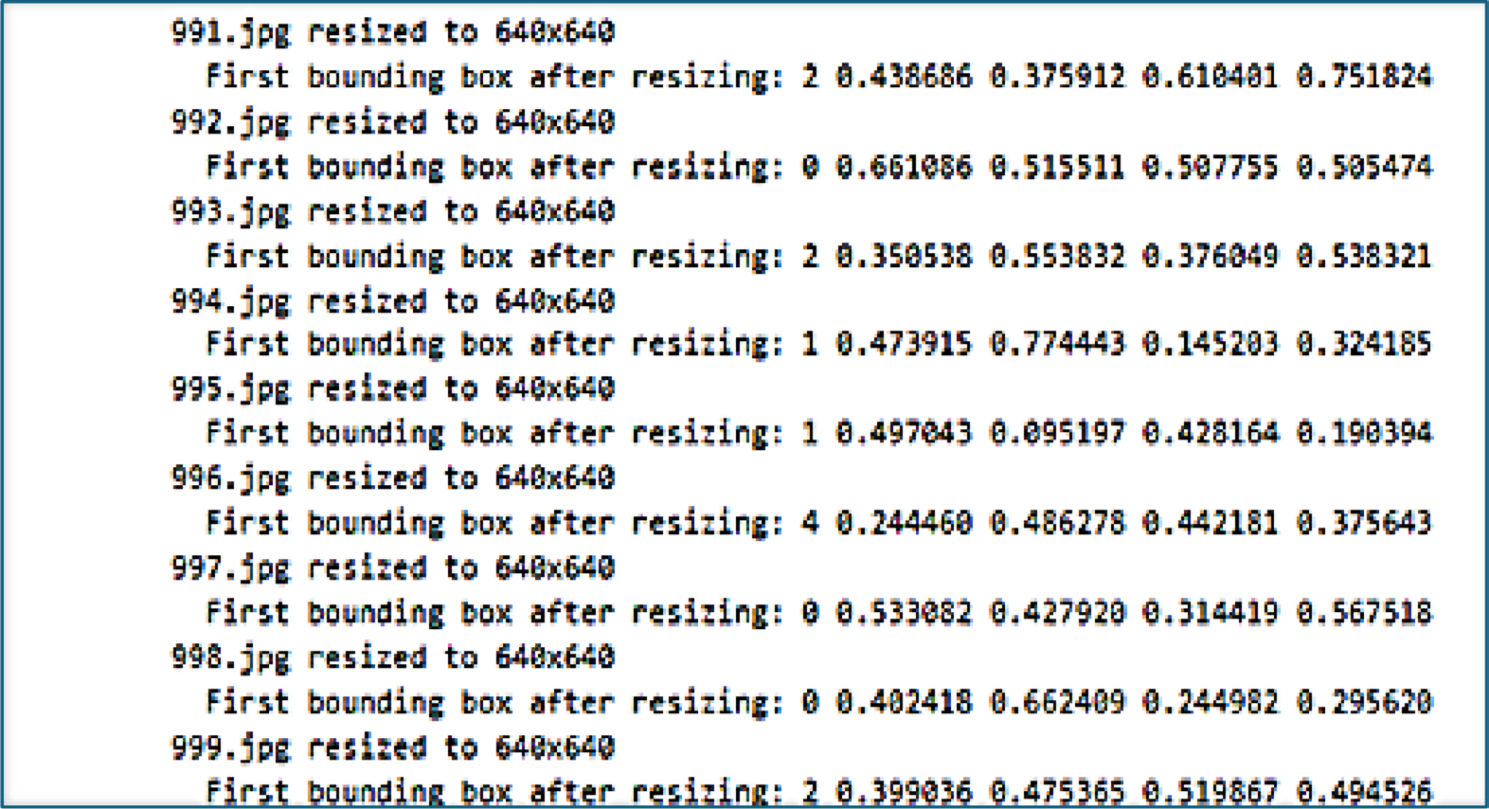
demonstrates the scaling of images at 640*640 and the bounding boxes corresponding to each image.

**Fig. 7:**
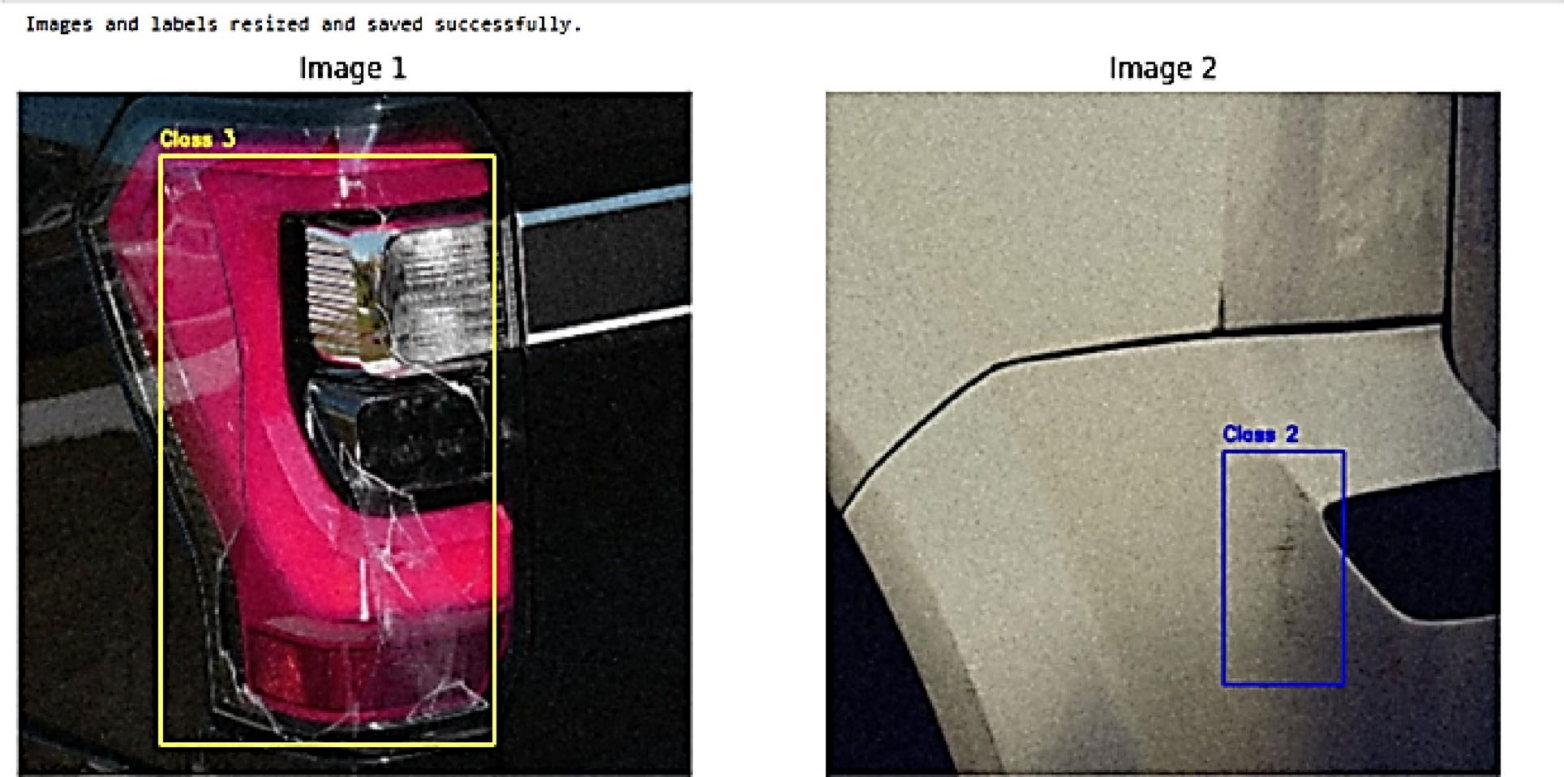
Illustrates the appearance of the images and bounding boxes after resizing.

Prior to the training, validation, and testing procedures of the model that is used to detect and classify damage, the pictures had to be resized to standardize their dimensions as part of the pre-processing steps required for the application. The 640*640-pixel picture size is the ideal choice as it strikes a compromise between size and resource efficiency, which is necessary to preserve details for detection. Applying the scaling procedure to the bounding box coordinates was also required, rather than just the pictures. This guarantees that the annotation and bounding boxes are accurate in relation to the picture dimensions, as presented in Figs. 6 and 7.

### Oversampling and data augmentation

**Fig. 8:**
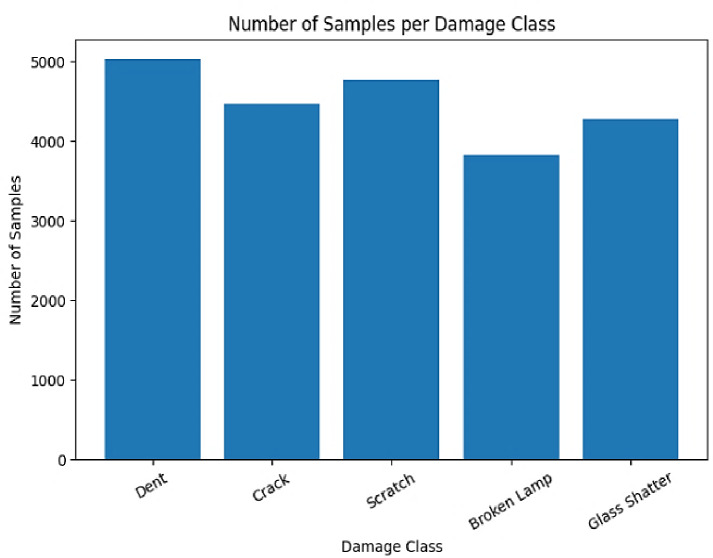
Illustrates the class distribution of the dataset after applying oversampling.

Since the initial data set revealed a significant disparity in the number of damage types, techniques were employed to raise the number to address the issue of imbalance in the original data between the categories for the damage types. The number of samples in each damage category was determined using annotation files in the yolo format, since the Broken Lamp and Glass Shatter categories had the lowest representation. It was calculated how many copies were required to increase the number of samples for each form of damage. Therefore, a specialized Python code was used to analyze the annotations and perform a census to determine the number of each category and the required number to achieve balance. After that, the images that contain the required categories are duplicated to increase. In addition, images are renamed using the shutil library to copy the images efficiently and accurately. Strategies were also created to modify the lighting, thus creating new examples and reducing overfitting. These strategies were used using the OpenCV library to ensure that the annotations are not affected by all these changes, and all of this led to achieving a balance between the classes, as presented in Fig. 8.

**Data augmentation**.

Data augmentation involves modified copies of an existing dataset; it is a technique that expands the dataset. Typical Techniques for Augmenting Data:

- Translation: Either move the picture horizontally or vertically to a certain distance.
- Scaling: Adjust the image’s dimensions by a predetermined amount.
- Flipping: the image may be flipped horizontally or vertically.
- Brightness: an augmentation approach, as changes to the photographs will change their basic composition.

Two techniques for data augmentation were employed in this study: horizontal flipping and brightness adjustment.

#### Brightness and Contrast Adjustment

The first method used to increase the amount of data while creating a difference between them is to modify the brightness and contrast. This modification was made using parameters to be suitable for the real conditions of lighting while maintaining the shape and form of the original image. As a result, each image’s lighting was modified by a linear transformation, as presented in Eq. 7^32^.

7 $$\:\mathbf{B}(\mathbf{m},\mathbf{n})=\mathbf{B}\_\mathbf{A}\mathbf{V}\mathbf{r}\:(\mathbf{m},\:\mathbf{n})\:(\mathbf{B}\_((\mathbf{m},\mathbf{n}))/(\mathbf{B}\_\mathbf{A}\mathbf{V}\mathbf{r}\:\:(\mathbf{m},\:\mathbf{n})\:\left)\right)^\mathbf{k}$$

Where:

B (m, n) is the brightness of a pixel with order number (m, n),

B̄_Avr (m, n) is the average across the region around the pixel (m, n),

K is a variable parameter.

To make the model able to generalize across this number of datasets, a scale factor (α) was drawn for each image between [0.7, 1.3], which is the best choice given that this range can reduce the contrast by 30%. The surface displacement factor (β) was also drawn from a distribution whose value ranges from − 40 to + 40.

**Fig. 9:**
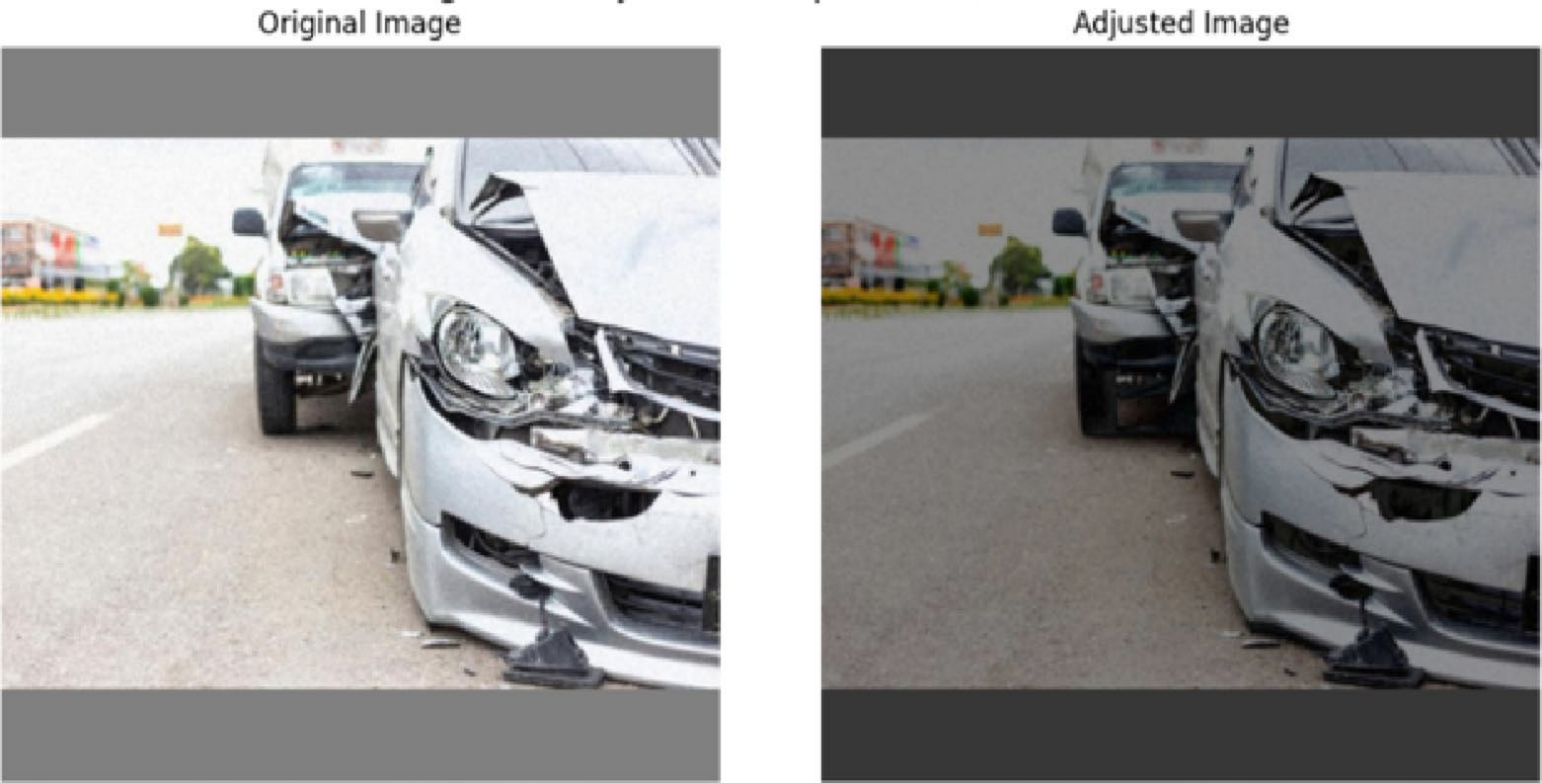
Illustrates the appearance of images after applying brightness and contrast adjustments.

This change in the degree of illumination led to an increase in the diversity of the data set more accurately and effectively, through the difference between the illumination without causing any defect or disturbance, thus improving the precision of the model to detect damage in different lighting conditions, as presented in Fig. 9.

#### Horizontal Flipping

**Fig. 10:**
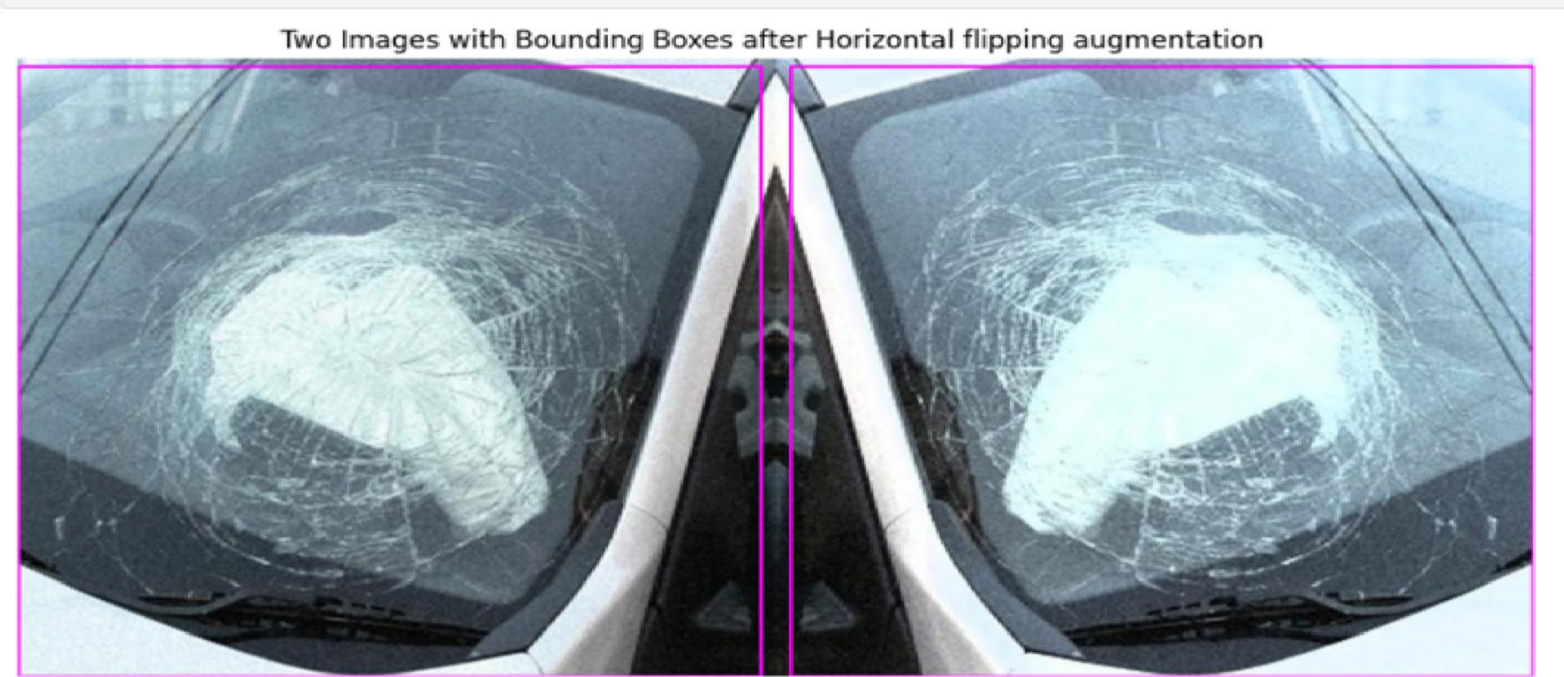
illustrates how photos look after being flipped horizontally.

The horizontal flipping approach was used to expand the data set’s size and diversity, which enhanced the variety of the damage categories that were underrepresented. A horizontal flip is the technique of flipping pictures horizontally to produce a mirror image of the original. This transformation flips the left and right sides of the image to create a mirror image effect. Vertical flipping was not used since it is impractical and causes faults in the picture and the accompanying bounding boxes, which forces the model to learn the inaccuracy. The technique of horizontal flipping was employed, in which the images were inverted with a probability of *p* = 0.5. This inversion produced reflection while preserving the bounding boxes and image diversity. The inversion was applied to the images as well as to the annotation, the type of damage, and the bounding box coordinates. The model is more accurate at detecting damage since the data set from the horizontal flipping operation shows the types of damage from the vehicles’ left and right sides, as presented in Fig. 10.

### Dataset splitting

The out-of-validation technique, also known as the hold-out validation method, is used to separate the data during the implementation. This is a popular technique for segmenting datasets in deep learning, particularly for classification, prediction, and damage detection. This helps to increase the model’s precision. It indicates that the data is divided into three sections: training, validation, and test data, rather than training and test data. The model is fitted using a training data set, which is a group of examples used to fit the parameters. The validation data set provides an evaluation of a model’s fit on the training data set while modifying the model’s hyperparameters without affecting the test data. The test data set is a set of information used to evaluate the effectiveness of the model to detect and classify vehicle damage based on the training data set. The dataset will be split into two phases at this point.

In the initial step, the dataset was partitioned in its original form without any modifications because it was imbalanced. The second step involves dividing the dataset before applying augmentation and oversampling procedures, which will balance the data (this includes Brightness adjustment, Flipping, Augmentation, and Oversampling).

#### Initial partitioning of the dataset

**Fig. 11:**
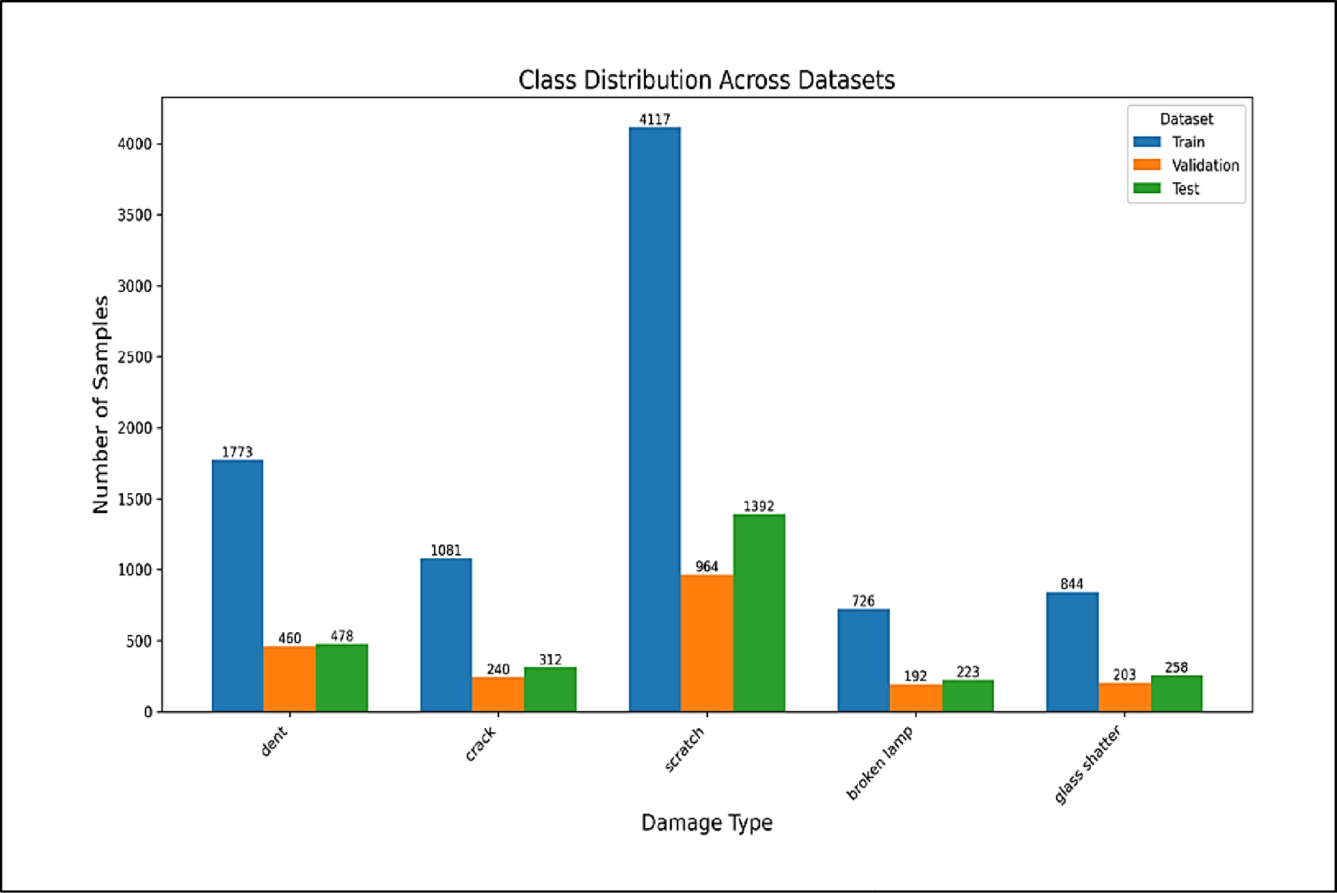
Illustrates the distribution of the imbalanced dataset after splitting.

The original dataset taken from Kaggle consisted of 6,666 images containing damaged vehicles. When labeling was done with bounding boxes, the dataset split ratios and the number of images contained in each split of the imbalanced dataset were as follows: 63.9% (4,260 images) for training, 16.1% (1,073 images) for validation, and 20.0% (1,333 images) for testing. To illustrate the distribution of each type of damage in the training, validation, and test splits of the imbalanced dataset is shown in Fig. 11.

#### Dataset distribution (Oversampling and data augmentation)

After partitioning the original dataset into training, validation, and testing subsets, oversampling and data augmentation were applied to avoid data leakage. Following these operations, the augmented training set contained 11,688 images, while the validation and test sets contained 2,468 and 2,224 images, respectively, resulting in a total of 16,380 images used during the experimental phase.

**Fig. 12:**
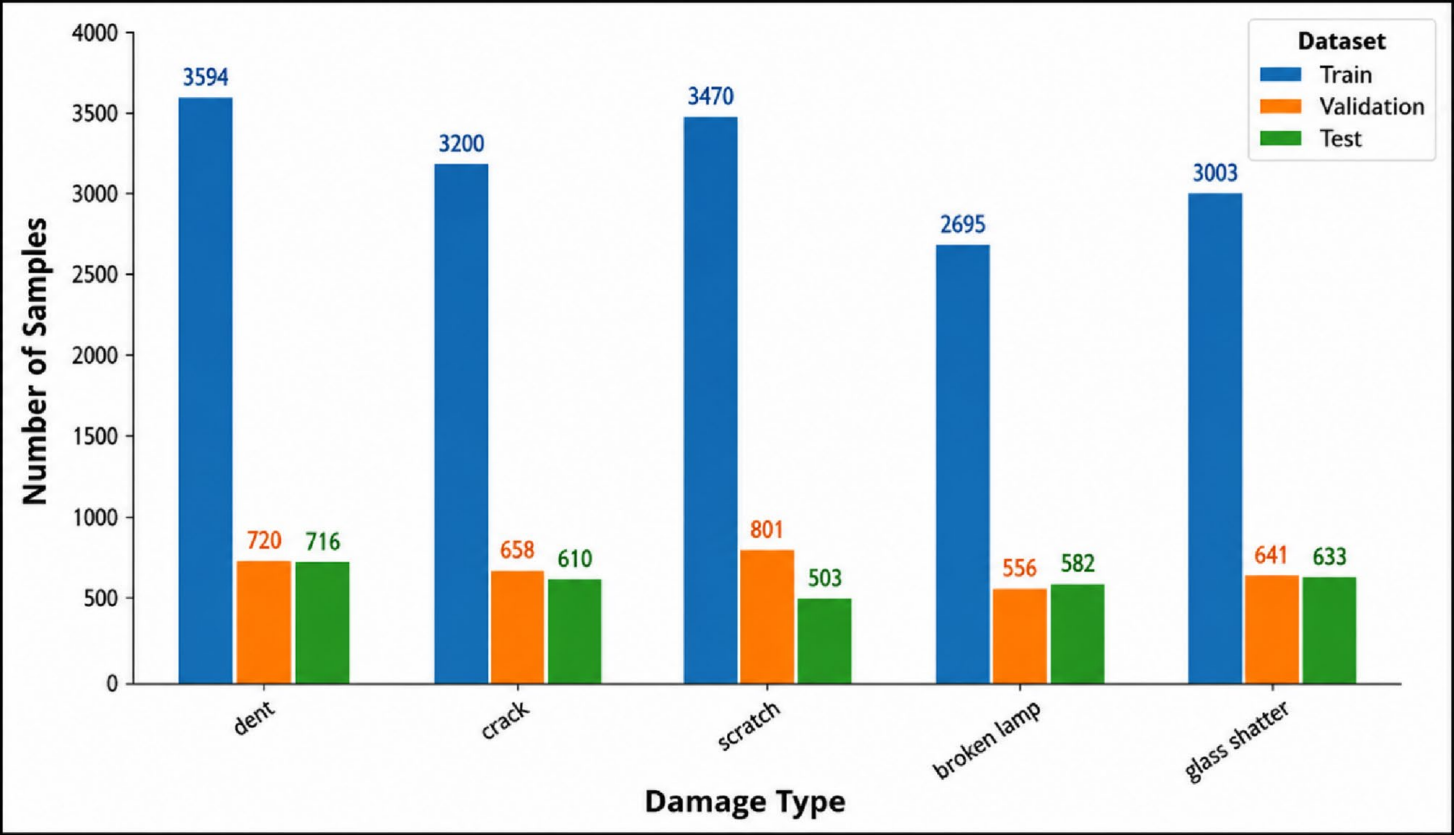
Illustrates the dataset distribution after Oversampling Augmentation(balancing).

This shows the final distribution of images in the balanced dataset. This distribution is for training, validation, and test sets as presented in Fig. 12.

### Building the cost dataset

In order to estimate the cost of each damage in each image accurately and effectively, and to build the cost associated with the dataset for detecting and classifying the damage, the cost dataset was built from scratch to ensure the integration of the purpose of the study on the same dataset with the help of experts in the field of repair. This is to determine realistic repair costs for five types of damage: dent, crack, scratch, broken lamp, and glass shatter. All of this had to be done from scratch because no publicly available dataset explained the costs of all five categories of damage.

After creating files containing data extracted from annotation files, metadata was added to them. This integration ensures that the dataset is richer and more useful for the model used to predict the cost. The metadata that was added is the expected cost of repair, the severity of the damage, and the location of the damage.

Two diverse sources were merged:

1. Annotation files: these files, which were created from scratch for all images for damage detection, extracted the important columns for use in cost prediction tasks.

These columns are:

#### Image name

This represents the name of the annotation file, as it is already named after the image.

**Damage type**: There are five types: crack, scratch, dent, broken lamp, and glass shatter. The damage type found in the annotation is extracted and placed in the damage type column.

**The bounding box coordinates consist of four numbers**,** which are**:

- X_center: This represents the horizontal center of the bounding box.
- Y_center: This represents the vertical center of the bounding box.
- Width: This represents the width of the bounding box.
- Length: This represents the height of the bounding box.

**2. A cost file and other metadata.** This file was created from scratch with the help of repair experts to generate data on the expected cost of repairing the damage, along with other important data.

The columns in this file are:

A. Image name.
B. Damage Type: This column represents the category of damage in the image.
C. Location: This column represents the location of the damage and is divided into three locations (front, side, rear).
D. Severity: This column represents the severity of the damage and is divided into three categories (low, medium, high).
E. Expected Cost: This column shows the expected cost of repairing this damage.

The image name and damage type were utilized to match the rows that relate to the same image with the same damage type in both files to finish the merging procedure between the files. The following were among the final columns that came from the merger process:

The cost dataset is split as presented in Table 3, showing the number of records available for each split.

**Table 3:** The Composition of the Cost Estimation Dataset after Splitting into Training and Validation Sets.

Dataset	Rows	Number of features
Train	13,720	9
Validation	3,022	
Test	3,113	8

### Cost data preprocessing

In this section, it will be discussed how to prepare a cost dataset and process it from scratch.

#### duplicate record elimination

Duplication will be eliminated in this phase to guarantee data integrity, following the successful merging of the files to produce the cost dataset. Based on the damage type and image name, duplicate rows were eliminated. This was done to eliminate any redundant annotations that would undoubtedly have an impact on the model during learning and prediction. The drop_duplicates function in the pandas’ package was used to implement the procedure of eliminating all duplicates. By eliminating any redundant remarks or mistakes made during the merging. Finally, the cost dataset was kept unique because it does not contain any duplication and ensures the integrity of the model during training and precision during testing.

#### Examination for missing values

After the merging step, the next step is to ensure the precision of the training and validation datasets to confirm their suitability and consistency. All columns were checked to search for any missing values. The check confirmed that there was no missing in the merged dataset, such as the coordinates of the bounding boxes, the type of damage, the type of damage, the location of the damage, the severity of the damage, or the cost of repair. This step was very necessary and important to ensure the integrity of the data before training because any incomplete data negatively affects the model learning process.

#### Label encoding

To guarantee compatibility with machine learning techniques, columns having textual values are transformed into numeric values at this point in the data preparation process. The cost dataset has three encoded classification columns. Damage type, severity, and location are listed in these columns.

- Damage Type Encoding:

Dent → 0.

Crack → 1.

Scratch → 2.

Broken Lamp → 3.

Glass Shatter → 4.

- Severity Encoding:

Low → 0.

Medium → 1.

High → 2.

- Location Encoding:

Front → 0.

Side → 1.

Rear → 2.

This step ensures a clear representation of textual variables, such as damage severity, and enables the model to understand and analyze the dataset consistently. The coding was done using Python code using the panda’s library.

To display the results, a part of the columns was printed after they were encrypted to ensure the correctness and consistency of the converted data. Finally, the columns were saved after encryption in an Excel file, and the old descriptive data were replaced with the data after encryption for use in training the model.

### Deep Learning for Vehicle Damage Detection and Classification

Following the preparation and pre-processing of the cost dataset, the critical phase of Deep Learning for Vehicle Damage Detection and Classification commences. This section introduces the application of deep learning, leveraging state-of-the-art object detection models to accurately detect and classify vehicle damage. This detailed analysis encompasses the model configuration, training performance, validation results, confusion matrix analysis, and the precision-recall curve analysis. As previously established, two distinct model architectures were utilized for damage detection and classification: YOLOv5 and YOLOv8. Both models were rigorously applied to both balanced and unbalanced datasets to assess performance under varying data distributions.

#### Model configuration

**For YOLOV5 (balanced dataset and imbalanced dataset)**.

Table 4 represents the YOLOv5 model’s size, image size used to train and validate the model, number of epochs, batch size, and type of optimizer used in the model.

**Table 4:** YOLOv5 configuration on balanced and imbalanced dataset.

Hyperparameters	values
Model	YOLOv5s
Image size	640 by 640 pixels.
epochs	100
Batch size	16
optimizer	Stochastic Gradient Descent (SGD)

**For YOLOV8 (balanced and imbalanced dataset)**.

Table 5 represents the YOLOv8 model’s size, image size used to train and validate the model, number of epochs, batch size, and type of optimizer used in the model.

**Table 5:** YOLOv8 configuration on the balanced and imbalanced dataset.

Hyperparameters	values
Model	YOLOv8n
Image size	640 by 640 pixels.
epochs	100
Batch size	16
optimizer	AdamW

#### Training performance

**Fig. 13:**
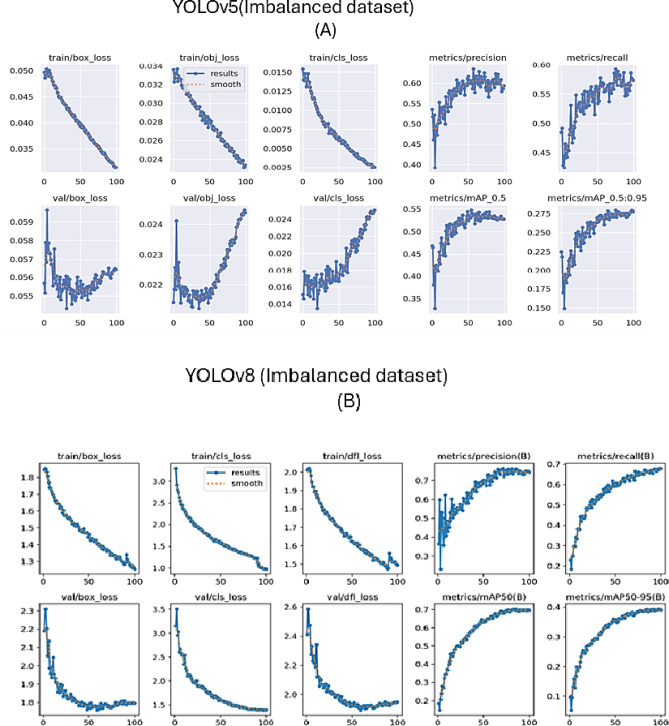

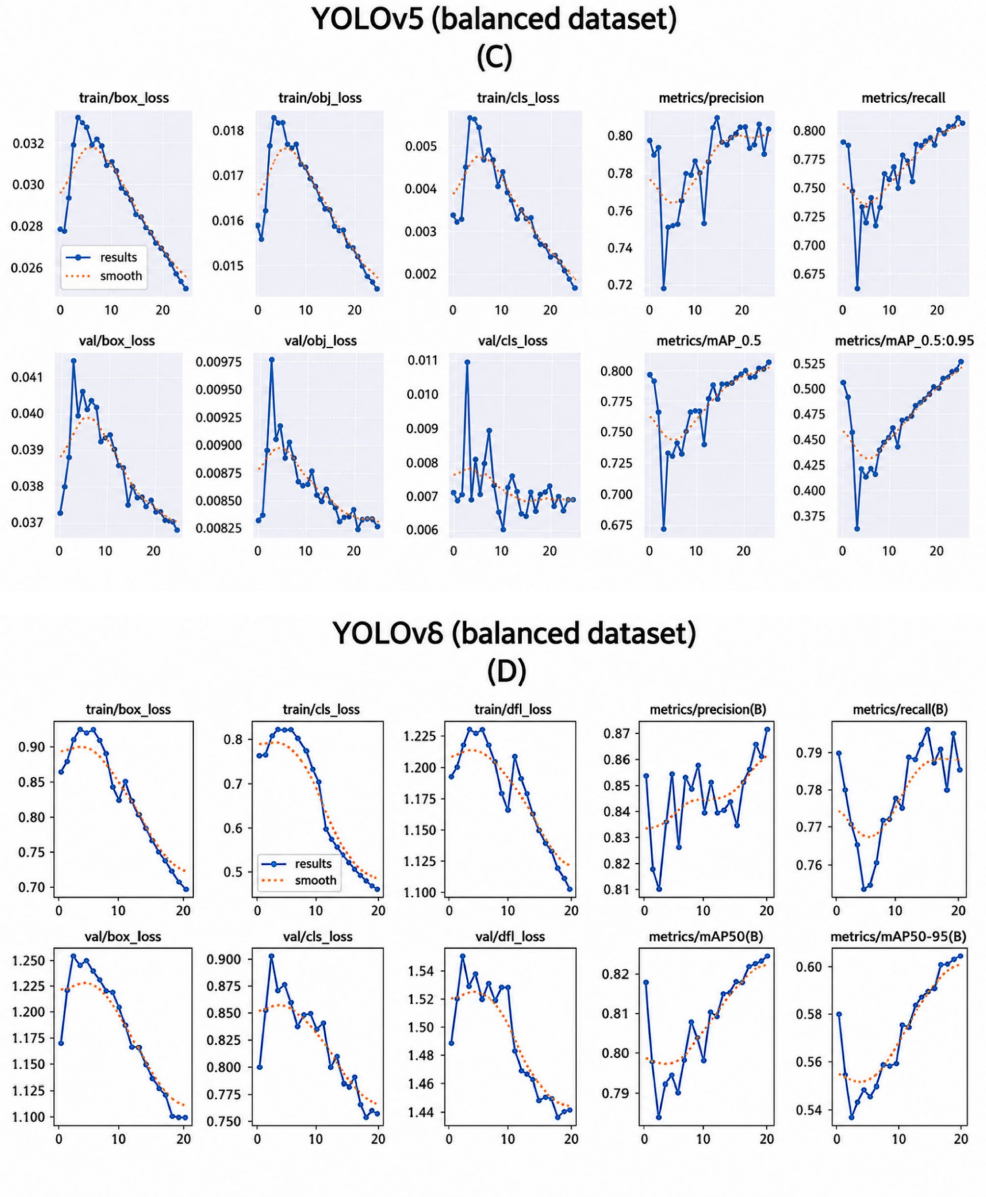
Illustrates the Training performance of YOLOv8 (on the imbalanced and the balanced dataset) and YOLOv5(on the imbalanced and the balanced dataset).

In this section, it will present the different metrics over the training epochs. Figure A represents the use of the YOLOv5 model on the imbalanced dataset, Figure B represents the use of the YOLOv8 model on the imbalanced dataset, and Figure C represents the use of the YOLOv5 model on the balanced dataset, Figure D represents the use of the YOLOv8 model on the balanced dataset, as presented in Fig. 13.

As shown in Fig. 13 (A), the YOLOv5 model used on an imbalanced dataset shows that the model retains training data for up to one hundred epochs by sloping downhill in a consistent manner.

According to the performance metrics, the validation metrics mAP@0.5 and mAP@0.5:0.95 show that the average precision is still poor, with mAP@0.5 stabilizing around 0.55. (metrics/precision, metrics/recall, metrics/mAP_0.5, metrics/mAP_0.5:0.95).

As shown in Fig. 13 (B), the YOLOv8 model trained on an imbalanced dataset showed a gradual decrease in both training and validation losses, starting from a high value of 1.6. The object loss also decreased noticeably; however, it exhibited significant fluctuations.

According to the performance metrics, both precision and recall increased gradually, but there was a noticeable lack of stability in the training process, as the final overall performance showed that the model was weak, with mAP@0.5 remaining below 0.5, and mAP@0.5:0.95 falling below 0.4.

As presented in Fig. 13 (C), the YOLOv5 model used on a balanced dataset showed extremely low values of loss as object losses are constant, indicating strong and stable learning.

According to the performance metrics, both precision and recall were good, but significantly lower than those of the YOLOv8 model used on a balanced dataset, where precision remained below 0.8. The mAP@0.5 reached around 0.78, while mAP@0.5:0.95 was around 0.5.

As shown in Fig. 13 (D), the YOLOv8 model used on a balanced dataset showed a very rapid decrease in all levels of losses, including train and validation losses and object losses. The losses decreased rapidly from around 0.8 to around 0.5, demonstrating the stability and effectiveness of the learning.

According to the performance metrics, both precision and recall improved significantly, reaching a value of around 0.8. The model achieved excellent results, with mAP@0.5 exceeding 0.85 and mAP@0.5:0.95 exceeding 0.6. This confirms the effective performance of the model and the effectiveness of the dataset balance.

#### Validation results

The YOLOv5 model ran for one hundred training epochs on the validation set, which consisted of 1,073 photos. Table 5 displays the matrix of the YOLOv5 model in an imbalanced dataset. Given that its recall of damages is 0.591, the YOLOv5 model’s precision of 0.593 suggests that it has a limited capacity to detect and classify damages, as presented in Table 6.

**Table 6:** Overall YOLOv5 Performance on an Unbalanced Dataset.

Metric	Value
Precision	0.593
Recall	0.591
mAP@0.5	0.544
mAP@0.5:0.95	0.28

Table 7 shows the per-class performance of YOLOv5 on the imbalanced dataset, including precision, recall, mAP@0.5, and mAP@0.5:0.95 for each type of damage. The model’s inability to recognize the five types of damage, particularly the dent, crack, and scratch categories.

**Table 7:** YOLOv5’s Performance per Class on Unbalanced Datasets.

Class	Precision	Recall	mAP@0.5	mAP@0.5:0.95
Dent	0.478	0.51	0.399	0.164
Crack	0.394	0.362	0.271	0.0941
Scratch	0.489	0.474	0.413	0.161
Broken Lamp	0.742	0.766	0.758	0.365
Glass Shatter	0.862	0.845	0.878	0.616

The YOLOv8 model ran for one hundred training epochs on the validation set, which consisted of 1,073 photos. Table 8 displays the matrix of the YOLOv8 model in an imbalanced dataset. Since the YOLOv8 model’s recall of damage is 0.678, this suggests that the model is still not exceptionally good at detecting damage, even though it reaches a precision of 0.749.

**Table 8:** Overall YOLOv8 Performance on an Unbalanced Dataset.

Metric	Value
Precision	0.749
Recall	0.678
mAP@0.5	0.698
mAP@0.5:0.95	0.393

Table 9 shows the per-class performance of YOLOv8 on the imbalanced dataset, which includes precision, recall, mAP@0.5, and mAP@0.5:0.95 for different forms of damage. This table demonstrates how well—though not flawlessly—the model detects five types of damage, particularly the shattered glass and damaged bulbs.

**Table 9:** YOLOv8’s Performance per Class on Unbalanced Datasets.

Class	Precision	Recall	mAP@0.5	mAP@0.5:0.95
Dent	0.683	0.6	0.61	0.282
Crack	0.657	0.527	0.537	0.212
Scratch	0.608	0.563	0.554	0.258
Broken Lamp	0.847	0.748	0.814	0.401
Glass Shatter	0.949	0.951	0.974	0.81

The validation set (2,468 photos) was run through one hundred epochs using the YOLOv5 model. Table 10 displays the matrix of the YOLOv5 model in a balanced dataset. Given that the YOLOv5 model’s recall of damage is 0.810, this suggests that the model is still effective in detecting and classifying damage, as seen by its precision of 0.806.

**Table 10:** The overall Performance of YOLOv5 on the balanced Dataset.

Metric	Value
Precision	0.806
Recall	0.810
mAP@0.5	0.803
mAP@0.5:0.95	0.522

Table 11 shows the per-class performance of YOLOv5 on the balanced dataset, which includes precision, recall, mAP@0.5, and mAP@0.5:0.95 for different forms of damage. The model’s ability to detect and classify the five types of damage.

**Table 11:** The Per-Class Performance of YOLOv5 on Balanced Dataset.

Class	Precision	Recall	mAP@0.5	mAP@0.5:0.95
Dent	0.730	0.758	0.697	0.372
Crack	0.750	0.787	0.786	0.485
Scratch	0.645	0.551	0.556	0.217
Broken Lamp	0.937	0.973	0.984	0.703
Glass Shatter	0.968	0.981	0.992	0.832

The validation set (2,468 photos) was subjected to one hundred epochs of the YOLOv8 model. Table 12 displays the matrix of the YOLOv8 model in a balanced dataset. Given that its recall of harm is 0.784, this suggests that the YOLOv8 model is quite good at detecting damage, as evidenced by its high precision of 0.87.

**Table 12:** The overall Performance of YOLOv8 on the balanced Dataset.

Metric	Value
Precision	0.87
Recall	0.784
mAP@0.5	0.824
mAP@0.5:0.95	0.606

Table 13 shows the per-class performance of YOLOv8 on the balanced dataset, which comprises precision, recall, mAP@0.5, and mAP@0.5:0.95 for different forms of damage. The YOLOv8 model’s superiority in detecting and classifying the five types of damage.

**Table 13:** The Per-Class Performance of YOLOv8 on Balanced Dataset.

Class	Precision	Recall	mAP@0.5	mAP@0.5:0.95
Dent	0.794	0.719	0.742	0.467
Crack	0.863	0.743	0.82	0.579
Scratch	0.744	0.532	0.586	0.257
Broken Lamp	0.967	0.953	0.983	0.811
Glass Shatter	0.982	0.975	0.991	0.916

#### Confusion Matrix Analysis

**Fig. 14:**
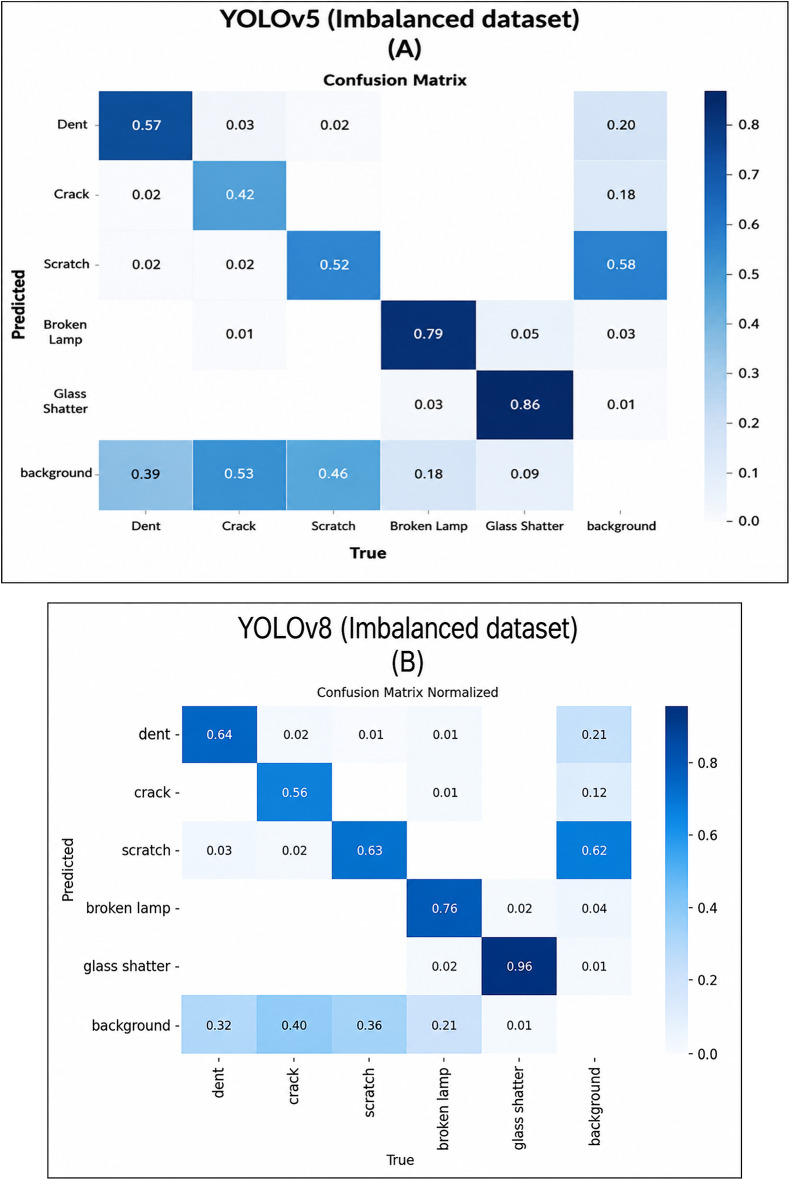

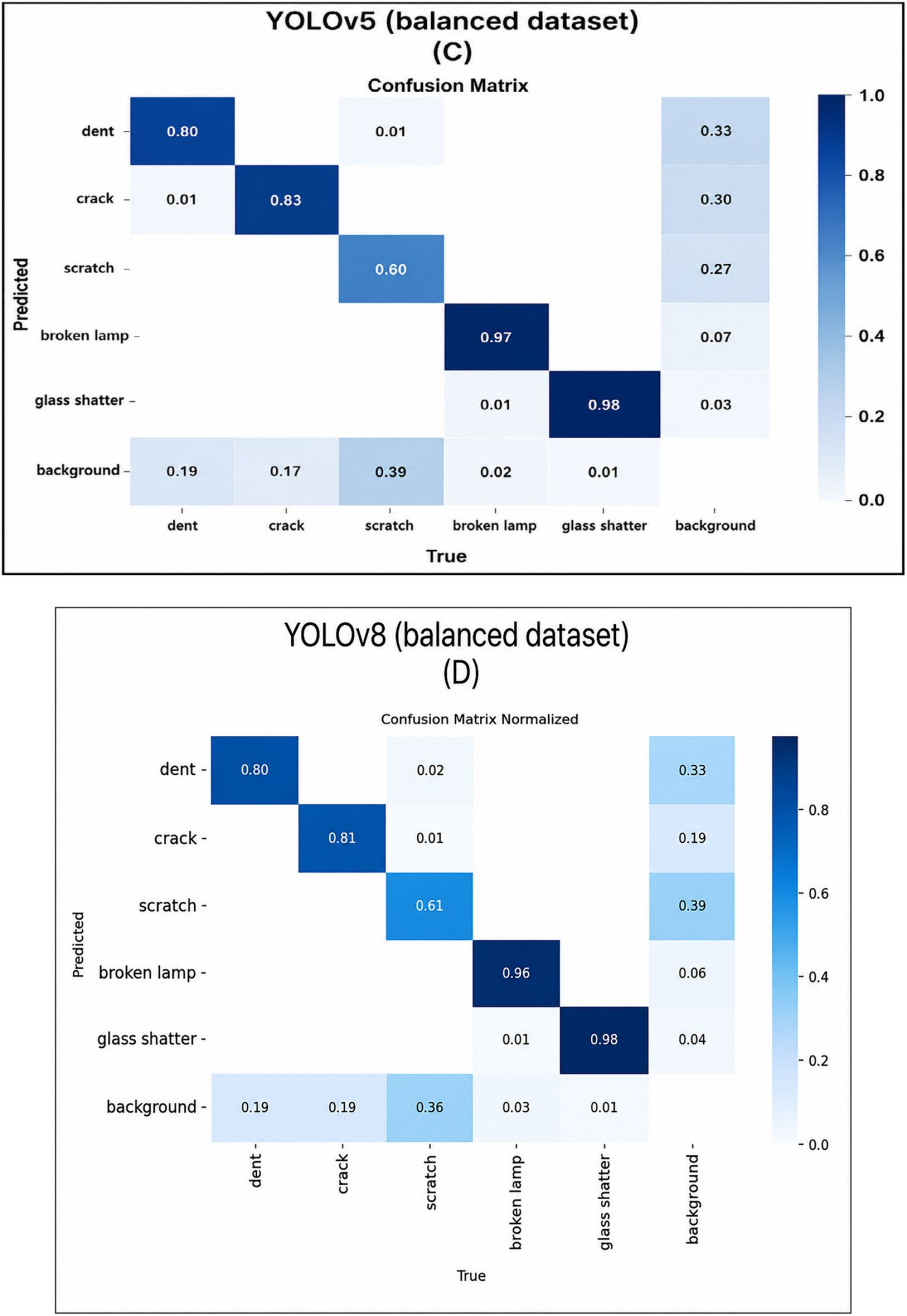
Confusion Matrix Analysis of YOLOv8 and YOLOv5 on Imbalanced and Balanced Datasets.

This section visualizes the classification errors between classes, as presented in Fig. 14.

As presented in Fig. 14 (A), YOLOv5 used on the imbalanced dataset suffers from its inability to detect types of damage, such as cracks. It achieves a low rate of correct detection in diverse types of damage.

Figure 14 (B) represents YOLOv8 used on the imbalanced dataset, showing low performance of around 0.6 and a recall of around 0.5, and suffers from unstable curves.

But Fig. 14 (C), using YOLOv5 on the balanced dataset, shows an overall precision of about 0.85 and a recall of about 0.75. Although these values are slightly lower than the performance of the YOLOv8 model, the YOLOv5 model demonstrates superior performance for categories such as ‘dent’ and ‘crack’ (between 0.80–0.97 and 0.60–0.83). However, performance is only average for categories such as ‘scratch’ (0.10–0.39) and ‘broken lamp’ (0.27–0.30), and poor for categories such as ‘glass shatter’ (0.31–0.34).

Figure 14 (D), YOLOv8 used on the balanced dataset. It shows much better results, with high precision of around 0.9 and a recall around 0.8. It also shows stability across different class types. The apparent distribution of all classes indicates balanced performance. The performance of the “broken lamp” class is excellent, as it achieved 0.96, while the results of the “dent” and “crack” classes are good because the results are (0.80) and (0.81). while the “scratch” class is reasonable as it is about (0.61).

#### Class-wise Performance Analysis

**Fig. 15:**
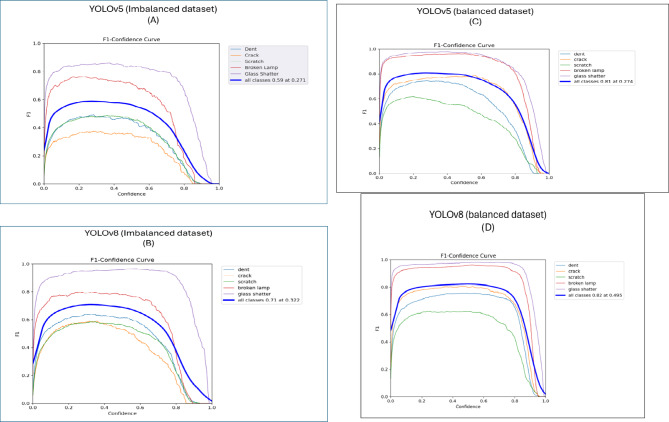
Illustrates class-wise performance analysis of YOLOv8 and YOLOv5 on Imbalanced and Balanced Datasets.

To gain a deeper focus on the model’s performance towards different damage types, the metrics for each model were analyzed by category, as presented in Fig. 15.

As presented in Fig. 15 (A), the F1 score curves for YOLOv5 used on the imbalanced dataset show the model’s ability to classify the distinct types of damage. For all damage categories, the highest F1 curve obtained from the F1-confidence curve is 0.59 at a 0.271 confidence level. Poor model performance in detecting and classifying damage is indicated by an F1 score of less than 0.6.

As presented in Fig. 15 (B), the F1 score curves for YOLOv8 used on the imbalanced dataset show significant variation across different damage types. Categories, such as “scratch” and “glass shatter,” have underrepresented F1 scores, indicating deficient performance for each type. The overall F1 score for all damage types is 0.77 with a confidence level of 0.322, which considered modest performance. The curve shapes are irregular, reflecting the model’s instability across all categories.

As presented in Fig. 15 (C), the F1 score curves for YOLOv5 used on the balanced dataset show that many classes exceed the F1 score of 0.8. However, there are classes, such as “scratch” and “glass shatter,” that show a decrease in the F1 score. This occurs with increasing confidence level, indicating a decrease in confidence. The maximum F1 score was 0.81 with a confidence of 0.274, but this indicates that it is lower than the score of the YOLOv8 model used on the balanced dataset.

As presented in Fig. 15 (D), the F1 score curves for YOLOv8 used on the balanced dataset achieve significantly higher F1 scores across all categories. Categories such as “crack,” “dent,” and “broken lamp” have strong F1 scores, above 0.8. The overall F1 score reaches its highest point at 0.92, with a confidence level of 0.495. This demonstrates the model’s robust performance at high confidence levels, and the curves appear smoother, indicating that the model is well-trained and stable.

#### Precision-Recall Curve Analysis

**Fig. 16:**
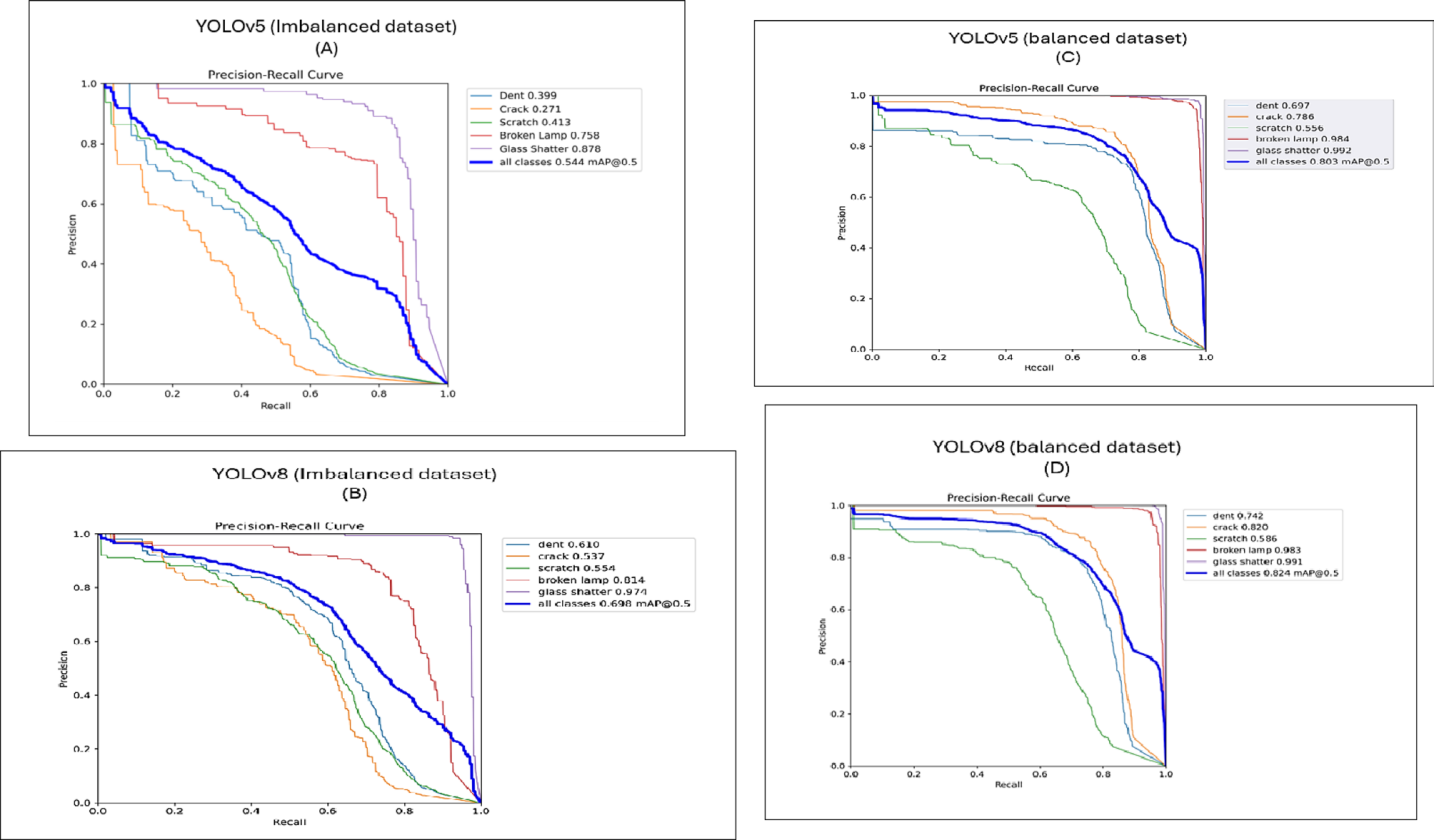
Illustrates the precision-recall curve of YOLOv8 and YOLOv5 on Imbalanced and Balanced Datasets.

In this section, the precision and recall curves for each model across different confidence levels are presented in Fig. 16.

As presented in Fig. 16 (A), the Precision-Recall (PR) curve for YOLOv5 was used on the imbalanced dataset. It indicates that it can identify the five types of damage with low precision, as seen by its average precision of 0.544. The precision-recall curve shows that the model can accurately detect glass shatters with an average precision of 0.878. Broken lights are the second most frequent type of damage after glass shattering, and the YOLOv5 model identified them with an average precision of 0.758. Other damage categories were identified by the model with deficient performance, including scratch damage (average precision of 0.413), dent category (average precision of 0.399), and fracture category (low average precision of 0.271). This implies that precision and curves are both decreasing fast.

As presented in Fig. 16 (B), the Precision-Recall (PR) curve for YOLOv8 used on the imbalanced dataset shows limitations. The overall performance is low, as evidenced by the decrease in precision with increasing recall values. This indicates false positives, and the curve indicates instability and low confidence.

As presented in Fig. 16 (C), the Precision-Recall (PR) curve for YOLOv5 was used on the balanced dataset. The results show that the overall performance is good, but there is a clear decline in some categories, such as “scratch” and “glass shatter”, where the maximum point reached by the curve is around 0.85 and the recall is close to 0.75, which indicates acceptable performance of the model.

As presented in Fig. 16 (D), the Precision-Recall (PR) curve for YOLOv8 used on the balanced dataset shows a strong and stable performance. The curve indicates high precision and recall, indicating that the performance is consistent. This indicates balanced learning, as the curve reached a maximum point of about 0.9 and recall of about 0.8, indicating the high reliability of the model when trained on a balanced dataset. It is ideal compared to YOLOv5 on the balanced dataset. According to the comparisons above, YOLOv8 on the balanced dataset has the best overall performance.

### Detecting the precision of the YOLOv8 model on the validation dataset

**Fig. 17:**
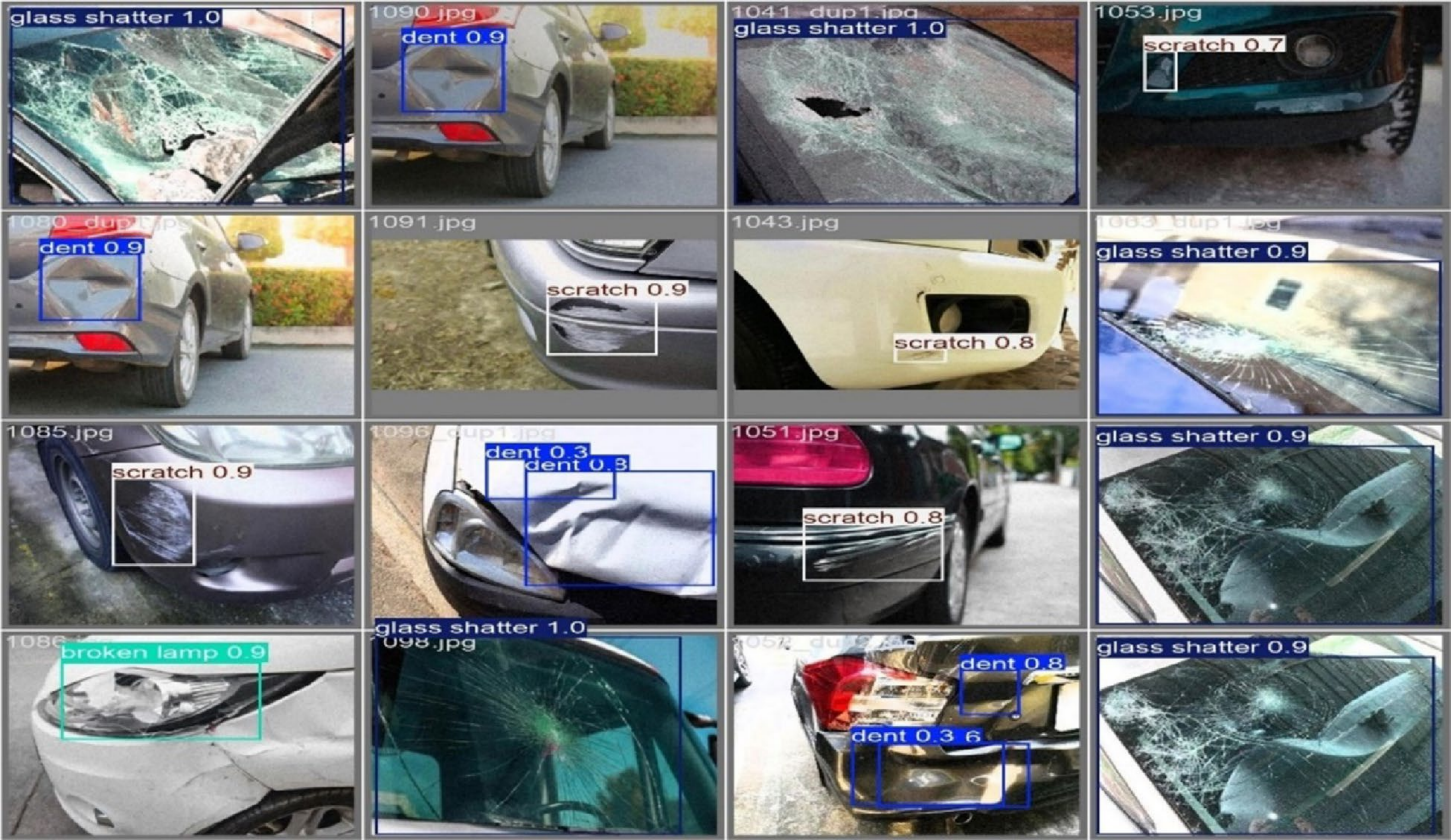
Illustrates the model’s results on the test images with confidence.

With a score ranging from 0.3 to 0.9 on the validation dataset of 2,468 photos, the YOLOv8 model demonstrated exceptional performance, demonstrating its ability to recognize a wide range of damage types, as presented in Fig. 17.

To further evaluate the object detection performance, the evaluation metrics of the YOLOv8 model trained on the balanced dataset has been presented. Table 14 summarizes the key performance indicators, including Precision, Recall, mAP@0.5, and mAP@0.5:0.95:

**Table 14:** Unified Evaluation Metrics (YOLOv8 - Balanced Dataset).

Metric	Definition	Value (Validation/Test)
Precision	Ratio of true positives to total predicted positives.	**0.870**
Recall	Ratio of true positives to all actual objects.	**0.784**
mAP@0.5	Mean Average Precision at 0.5 IoU threshold.	**0.824**
mAP@0.5:0.95	Average mAP calculated over IoU thresholds from 0.5 to 0.95.	**0.606**

### YOLOv8 Model Performance on Test Images

**Fig. 18:**
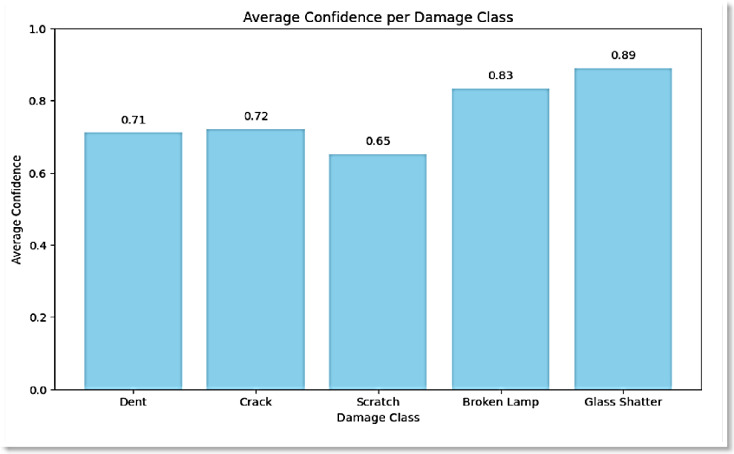
Illustrates a bar Chart Showing the Average Confidence per Damage Class.

This section evaluates the YOLOv8 model under various conditions using the test dataset of 2,224 photos. First, the YOLOv8 model performed exceptionally well on the 2,224-image test dataset, with an average confidence between 0.65 and 0.89 and a score between 0.65 and 0.95, demonstrating the model’s ability to accurately recognize different forms of damage, as presented in Fig. 18.

Second, the YOLOv8 model’s capacity to recognize distinct types of damage in a single image is crucial for vehicle evaluation. Since it is highly likely that a single vehicle would sustain various sorts of damage, insurance companies, and repair businesses demand this. Lastly, it was demonstrated that the model could successfully detect and classify damage when evaluated on images in various lighting scenarios.

### Comparing model results on test data with actual data

**Fig. 19:**
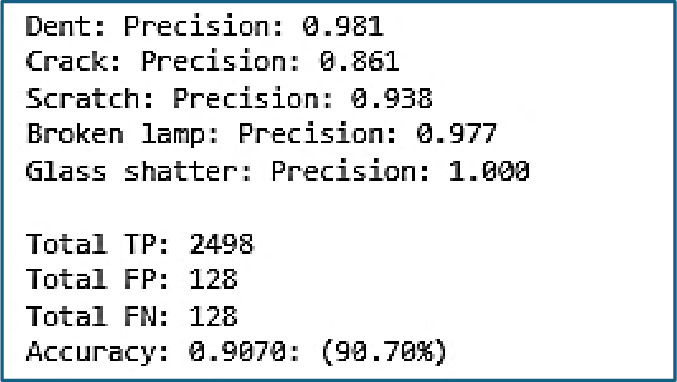
Illustrates the results from comparing model results on test data with actual data.

When contrasting the actual bounding boxes and damage types created from scratch on the test dataset with those generated by evaluating the YOLOv8 model on the test dataset. The findings showed that the precision of the dent was 0.981, the precision of the crack was 0.861, the precision of the scratch was 0.938, the precision of the broken light was 0.977, and the precision of the glass shatter was 1.000, as presented in Fig. 19.

### Machine learning for Repair Cost Prediction

The XGBoost model was selected because it is among the most sophisticated models for cost prediction, particularly for repair costs. Consequently, after precisely detecting and classifying the damage, this model will assist in predicting the cost of repairs for vehicle damage. The annotation files were used to extract these characteristics. These are the bounding box coordinates and the types of damage. The severity of the damage, which is divided into three categories—low, medium, and high—the position of the damage, which is divided into three categories—front, side, and rear—and the expenses of fixing each damage independently are additional elements that were introduced.

The hyperparameters and the values used for each hyperparameter in the XGBoost model are presented in Table 15.

**Table 15:** shows the hyperparameters with their values used when training the XGBoost model.

Hyperparameter	Value
subsample	0.5
n_estimators	100
min_child_weight	8
max_depth	3
learning_rate (η)	0.05
colsample_bytree	0.5
gamma	0.2

Comparative context is essential to justify the selection of the XGBoost model. To address this issue, an extensive benchmark study comparing XGBoost against three standard regression baselines: linear regression, support vector regression (SVR), and random forest regressor are conducted as presented in Table 16.

**Table 16:** Comparative Analysis of Regression Models for Cost Prediction.

Model	*R*² Score	MAE (EGP)
Linear Regression	0.4769	644.87
Support Vector Regression (SVR)	0.6111	465.74
Random Forest Regressor	0.7526	305.06
XGBoost (Proposed)	**0.9728**	**128.50**

The comparative analysis demonstrates that XGBoost is the most effective model for repair cost prediction, achieving a superior R² of 0.9728 and the lowest MAE (128.50 EGP). It significantly outperformed Random Forest (R²=0.75), SVR (R²=0.61), and Linear Regression (R²=0.47). XGBoost’s gradient boosting architecture successfully captures the complex, non-linear relationships between damage geometry and expert-validated costs that traditional models like SVR struggled to handle. This high precision justifies its selection as the primary engine for our framework, ensuring reliable financial estimations.

### Model performance

The performance of the XGBoost model has been evaluated using the following criteria, which are:

- R-squared: 0.9728.
- Mean Absolute Error (MAE): 128.50.
- MAE as % of mean cost: 7.66%.

The XGBoost model has outstanding predictive performance, as evidenced by the R^2^ ratio, which shows that it can represent 97.28% of the car repair cost predictions. The model produces an average absolute error of 128.50. According to the MAE value, this means that the margin of error is 7.66%.

**Fig. 20:**
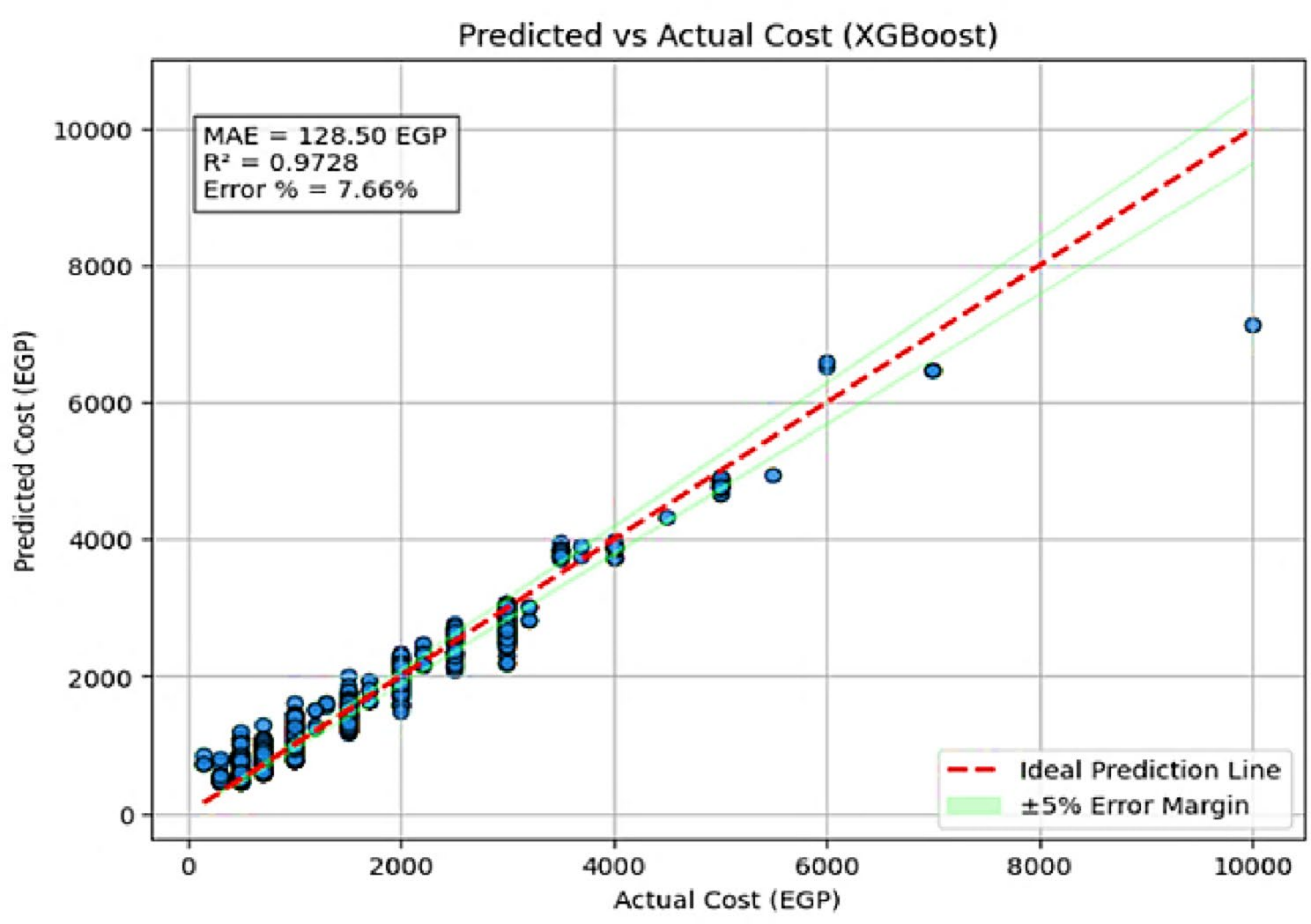
demonstrates the predicted versus actual Cost.

With 97.28% of the predictions falling within a 7.66% error range, the scatter chart demonstrates that the XGBoost model predicts auto repair expenses with exceptional accuracy. The red dashed line, which represents the perfect prediction and shows that the actual and anticipated values are near one another, is near the blue dots. Other measures include MAE, which equals 128.50, and R2, which reaches 0.9728. These measurements show the model’s accuracy at cost levels ranging from 2,000 to 10,000. It displays the model’s accuracy in terms of actual cost, as presented in Fig. 20.

**Fig. 21:**
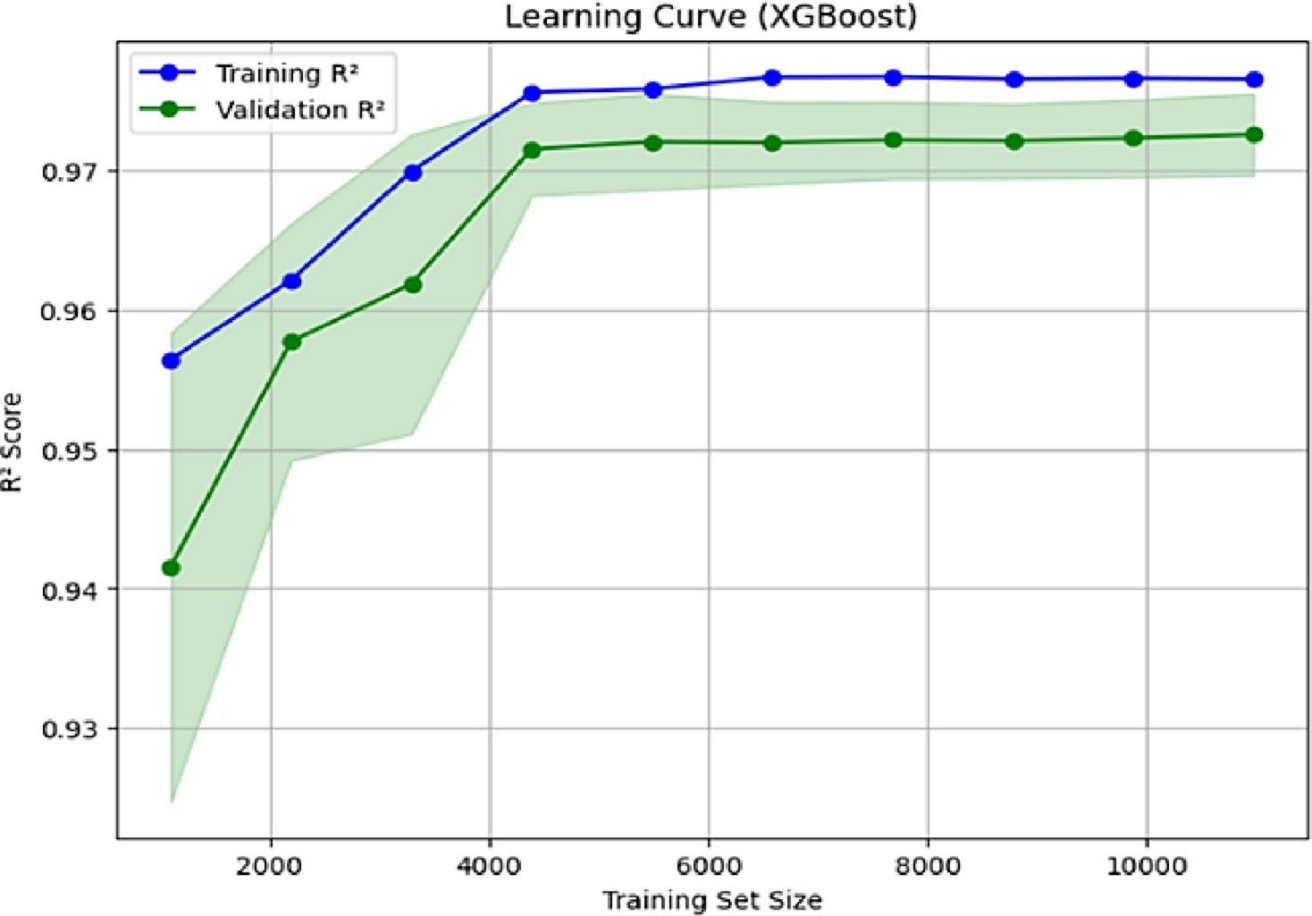
demonstrates the Learning Curve of the XGBoost model.

The learning curve of the XGBoost model demonstrates its outstanding performance. The model performs well on the training data, as seen by the blue training R2 curve, which is near 0.978 at all dataset levels. At low dataset levels, the green validation R2 curve is low and occasionally erratic, but as the dataset grows, it eventually hits 0.9728. This tiny difference in the model’s performance on the training and validation data shows that overfitting was avoided, demonstrating the model’s ability to generalize well to previously unseen data. The green area decreases with increasing training data size, as presented in Fig. 21.

### Discussion

The objective of the CarDD study^33^, which employed the same dataset with the same kinds of damages as this suggested framework, was to detect and classify damage exclusively and cease offering insurance companies ways to resolve insurance claims. Nonetheless, our suggested framework offers a complete solution that predicts repair costs in addition to detecting and classifying defects.

The CarDD study’s dataset was unbalanced, and no effort was made to make it more balanced. However, in this suggested framework, the unbalanced data was used, techniques were applied to make it more balanced, and object detection models were applied to both types of data to compare their precision.

Different models were used for the algorithms used in the CarDD study, but DCN+ with ResNet-101 was the most accurate model, achieving a precision of 60.6%. Its precision in each damage category was as follows: dent equals 42.2, scratch equals 42.3, crack equals 29.6, shattered glass equals 90.1, and broken lamp equals 69.5. However, YOLOv5 was applied to imbalanced data in this suggested framework and obtained a precision of 0.593. Its precision in each damage category was 0.478 for dents, 0.489 for scratches, 0.394 for cracks, 0.862 for shattered glass, and 0.742 for broken lamps.

However, YOLOv5’s precision on the balanced dataset was 0.806, and it was 0.730 for dents, 0.645 for scratches, 0.750 for cracks, 0.968 for shattered glass, and 0.937 for broken lamps. While the YOLOv8 model’s precision on imbalanced data was 0.749, it was 0.683 for dents, 0.608 for scratches, 0.657 for cracks, 0.949 for smashed glass, and 0.847 for broken lamps. However, YOLOv8’s precision on balanced data was 0.87, and it was 0.794 for dents, 0.744 for scratches, 0.863 for cracks, 0.982 for glass shatters, and 0.967 for broken lamps. As shown in Table 17, it was demonstrated that YOLOv8’s precision on balanced data is superior to that of the earlier research.

**Table 17:** Comparing the results of the proposed framework with another study.

Paper	Purpose			Dataset	Algorithm	precision
	Detection	Classification	Cost			
^20^	✓	✓	✖	Imbalanced dataset	DCN+ with ResNet-101	60.6%.
Proposed Framework	✓	✓	✓	Imbalanced dataset	YOLOv5	59.3%
					YOLOv8	74.9%
				balanced dataset	YOLOv5	80.6%
					YOLOv8	87%

Compared to the study^20^, The suggested framework is more accurate. This is because of two crucial factors: First, methods for balancing the data have a beneficial and useful impact on increasing precision. The second is that the suggested framework in this study is more suited for scientific application since it uses simpler and quicker models than the CarDD study’s models, which employ slower and more complicated models.

A critical issue becomes evident upon reviewing prior research: most existing studies do not provide a fully integrated solution to support insurance companies in vehicle damage detection, classification, and repair–cost prediction. As summarized in Table 18, most of the literature addresses only a single aspect of the vehicle assessment process rather than approaching it holistically.

**Table 18:** Matrix of Machine Learning and Deep Learning Techniques for Car Damage Analysis.

Techniques	Machine Learning (ML)			Deep learning (DL)		
Purpose	Detection	Classification	Cost	Detection	Classification	Cost
^34^	✓ (ML)			✖ (DL)		
	✖	Classification	Cost	✖	✖	✖
^35^	✓ (ML)			✖ (DL)		
	✖	✖	Cost	✖	✖	✖
^36^	✖ (ML)			✓ (DL)		
	✖	✖	✖	Detection	Classification	✖
^37^	✖(ML)			✓ (DL)		
	✖	✖	✖	✖	Classification	✖
^38^	✖(ML)			✓(DL)		
	✖	✖	✖	✖	Classification	✖
^39^	✖(ML)			✓ (DL)		
	✖	✖	✖	✖	Classification	✖
^40^	✖(ML)			✓ (DL)		
	✖	✖	✖	✖	Classification	✖
^41^	✖(ML)			✓(DL)		
	✖	✖	✖	✖	Classification	✖
^42^	✖(ML)			✓ (DL)		
	✖	✖	✖	✖	Classification	✖
^43^	✖(ML)			✓ (DL)		
	✖	✖	✖	Detection	Classification	✖
^44^	✖(ML)			✓ (DL)		
	✖	✖	✖	Detection	Classification	✖
^45^	✖(ML)			✓ (DL)		
	✖	✖	✖	Detection	Classification	✖
^46^	✖(ML)			✓ (DL)		
	✖	✖	✖	Detection	Classification	✖
^47^	✖(ML)			✓(DL)		
	✖	✖	✖	Detection	Classification	✖
^48^	✓ (ML)			✖(DL)		
	✖	✖	Cost	✖	✖	✖

## Conclusion and future work

The traditional methods for this type of review require time, energy, and skilled human labor, and they lead to bias in the evaluation, which results in inaccurate insurance claims processing and unhappy clients of insurance firms.

This paper presents an integrated framework between deep learning and machine learning for vehicle damage assessment. It combines YOLOv8 for damage detection, classification, and XGBoost for cost prediction. Finding a dataset dedicated to detection, classification damage as well as predicting repair costs is the first step. However, it was discovered that this data was limited, and all that was needed to employ deep learning and machine learning techniques was to concentrate on creating the dataset from scratch. The dataset, which includes images of damaged vehicles, was gathered via Kaggle. The cost dataset is constructed using the dataset for damage detection and classification, and an annotation file is created for every picture so that the model may be trained on them.

To detect and classify the damage, deep learning techniques were used, especially the YOLOv5 and YOLOv8 versions. They are fast and accurate, so they were chosen for damage detection and classification tasks. Different versions with different optimizers were used and applied to two types of data. The first type was an unbalanced dataset, and the second was a balanced dataset, so the precision difference could be seen.

When the YOLOv5 model was used on the imbalanced dataset, it achieved Precision 0.593, Recall 0.591, mAP@0.5 0.544, and mAP@0.5 0.95 0.28. When the YOLOv5 model was used on the balanced dataset, it achieved Precision 0.806, Recall 0.810, mAP@0.5 0.803, and mAP@0.5 0.95 0.522.

When the YOLOv8 model was used on the imbalanced dataset, it achieved Precision 0.749, Recall 0.678, mAP@0.5 0.698, and mAP@0.5 0.95 0.393, but when the YOLOv8 model was used on the balanced dataset, it achieved Precision 0.87, Recall 0.87, mAP@0.5 0.824, and mAP@0.5 0.95 0.606. It is clear from all these values of matrices that the reason for the increased precision is due to making the data more balanced than before.

Based on these results, the YOLOv8 model demonstrated its effectiveness on the balanced dataset, so it was therefore used on test data. The model’s results from detecting and classifying are compared with actual datasets built from scratch. The model achieved a precision of 90.7%, which indicates the effectiveness of this model in detecting and classifying damage.

For the cost dataset created from scratch, an insurance expert was consulted to ensure data accuracy and reliability for application in this study. The XGBoost model is used to train and validate this data set to predict the cost of repairing damage. The model achieved a Mean Absolute Error (MAE) of 128.50 and an R² score of 0.9728 on the validation set.

### Future work

Several potential directions for future research are identified to further enhance vehicle damage detection, classification, and repair cost prediction. These enhancements are expected to improve predictive accuracy and strengthen the overall assessment process:

First, Future work could expand the damage categories beyond the existing five categories included in this study: scratches, cracks, dents, broken lamps, and shattered glass. This allows for the detection of wider types of damage. Automated assessment can become more accurate with images containing types of damage that are not visible or have not been previously trained on.

Second, future developments of this dataset may include tasks beyond classification and detection. Segmentation tasks could also be added, allowing the model to detect damage not only through bounding boxes but also by the exact shape and region of the damage. This would enable a more accurate and detailed representation of each damaged instance.

Finally, there is an urgent need to build models capable of classifying the severity of vehicle damage. Such models would contribute to more accurate and reliable repair-cost predictions, improving decision-making for stakeholders such as insurance companies and repair services.

## Data Availability

The datasets generated during and/or analyzed during the current study are available from the corresponding author on reasonable request.
